# The micro-RNA content of unsorted cryopreserved bovine sperm and its relation to the fertility of sperm after sex-sorting

**DOI:** 10.1186/s12864-020-07280-9

**Published:** 2021-01-07

**Authors:** Esin Keles, Eleni Malama, Siyka Bozukova, Mathias Siuda, Sarah Wyck, Ulrich Witschi, Stefan Bauersachs, Heinrich Bollwein

**Affiliations:** 1 Clinic of Reproductive Medicine, Vetsuisse Faculty, University of Zurich, CH-8057 Zurich, Switzerland; 2 Veterinary Research Institute, Hellenic Agricultural Organization Demeter, 57001 Thermi, Thessaloniki Greece; 3 Institute of Veterinary Anatomy, Vetsuisse Faculty, University of Zurich, CH-8057 Zurich, Switzerland; 4 Swissgenetics, CH-3052 Zollikofen, Switzerland

**Keywords:** sex-sorted sperm, microRNA, miRNA, small RNA-seq, bull fertility

## Abstract

**Background:**

The use of sex-sorted sperm in cattle assisted reproduction is constantly increasing. However, sperm fertility can substantially differ between unsorted (conventional) and sex-sorted semen batches of the same sire. Sperm microRNAs (miRNA) have been suggested as promising biomarkers of bull fertility the last years. In this study, we hypothesized that the miRNA profile of cryopreserved conventional sperm is related to bull fertility after artificial insemination with X-bearing sperm. For this purpose, we analyzed the miRNA profile of 18 conventional sperm samples obtained from nine high- (HF) and nine low-fertility (LF) bulls that were contemporaneously used to produce conventional and sex-sorted semen batches. The annual 56-day non-return rate for each semen type (NRR_conv_ and NRR_ss_, respectively) was recorded for each bull.

**Results:**

In total, 85 miRNAs were detected. MiR-34b-3p and miR-100-5p were the two most highly expressed miRNAs with their relative abundance reaching 30% in total. MiR-10a-5p and miR-9-5p were differentially expressed in LF and HF samples (false discovery rate < 10%). The expression levels of miR-9-5p, miR-34c, miR-423-5p, miR-449a, miR-5193-5p, miR-1246, miR-2483-5p, miR-92a, miR-21–5p were significantly correlated to NRR_ss_ but not to NRR_conv_. Based on robust regression analysis, miR-34c, miR-7859 and miR-342 showed the highest contribution to the prediction of NRR_ss_.

**Conclusions:**

A set of miRNAs detected in conventionally produced semen batches were linked to the fertilizing potential of bovine sperm after sex-sorting. These miRNAs should be further evaluated as potential biomarkers of a sire’s suitability for the production of sex-sorted sperm.

**Supplementary Information:**

The online version contains supplementary material available at 10.1186/s12864-020-07280-9.

## Background

Manipulating the calf sex ratio can be a powerful tool for increasing the profitability and for accelerating the genetic gain in dairy and beef cattle farming [1–3]. Thus, it is not a surprise that the use of sex-sorted sperm in bovine assisted reproduction has steadily increased in the last years [4, 5]. Although alternative methodologies have been described [6–8], the separation of X- and Y-bearing spermatozoa by means of flow cytometry after Hoechst 33342 labeling is still the technique of choice applied in most sorting facilities, mainly due to its high accuracy, repeatability and suitability for commercial application [9]. Nevertheless, several research groups had already reported that inseminating dairy heifers with a dose of 1 to 2 million frozen-thawed X-bearing sperm resulted in conception rates not higher than 70–90% of these achieved with unsorted sperm (henceforward mentioned as “conventional” in the text; [10–15]). Consequently, along with the higher price of sex-sorted products, a variable loss in bull fertility appeared to be the major cost of artificial insemination (AI) with sex-sorted sperm [16, 17] and, thus, a considerable drawback to the global expansion of its use.

Recent advancements in sorting technology in combination with an almost two-fold increase of the number of sperm per AI dose (i.e. 4 instead of 2.1 million sex-sorted sperm per dose) are expected to address the fertility problem both in heifers and cows, resulting in non-return rate (NRR) values of approximately 90% of those obtained after AI with conventional sperm [18–20]. Nonetheless, the production of sex-sorted sperm remains an expensive procedure and processing ejaculates of sires that do not perform optimally after sex-sorting costs a considerable amount of resources. Post-thaw quality characteristics of sex-sorted sperm can be of some predictive value for its fertilizing potential after AI [21]; however, this information is available only at late stages of the production process, when sire and ejaculate selection, semen logistics, sperm sorting and cryopreservation, all time-consuming and costly procedures, have already taken place. Not surprisingly, the NRR for conventional semen (NRR_conv_) has not been proven a reliable indicator of the NRR for sex-sorted semen (NRR_ss_) either, even when equal doses of both semen types were used for the generation of NRR data [22, 23]. Indeed, a large study on dairy bulls used for the production of both conventional and sex-sorted sperm in the U.S.A. demonstrated that sire fertility rankings significantly differ between the two semen types [24].

Several studies have shown that the fertilizing potential of sperm after sorting largely varies between bulls when used either for field AI [12, 20, 23, 25, 26] or for in vitro embryo production [27–29]. It is well known that NRR values respond to increasing sperm doses in a bull-dependent manner; this response pattern is linked to the level of non-compensable defects present in sperm and has been documented for both conventional [30, 31] and sex-sorted sperm [25]. There is also indication that sperm tolerance to mechanical stress (i.e. sorting pressure) and prolonged storage prior to sorting varies between individuals [12]. Interestingly, sex-sorting affects sperm molecular mechanisms in a bull-dependent manner too. In a split-ejaculate experiment, Carvalho et al. (2012) observed that the effects of the sorting procedure on the methylation profile of the *IGF2R* gene of Y-bearing sperm differed significantly between bulls [32]. Thus, a better understanding of bull-specific factors that affect the functional status and molecular biology of sperm cells after sorting would substantially contribute to fertility prognostics of sex-sorted sperm [9, 33].

Studies about the impact of sex-sorting on the molecular features of sperm and the respective consequences for male fertility are rather scarce. It has been shown that both sex-sorting and cryopreservation induce epigenetic changes to sperm, particularly related to their gene methylation pattern [32] and transcriptome profile [34, 35]. In the same direction, Morton et al. (2007) described differences in the relative transcript abundance of developmentally relevant genes between day-7 bovine embryos that were in vitro produced using conventional and sex-sorted sperm [36]. Similar findings have also been reported in other ruminant species [37]. The authors attributed the differential expression of these embryonic genes to alterations of sperm molecular characteristics after sex-sorting; however, the nature of these alterations was not further investigated [36].

Among other RNA molecules, small non-coding RNAs (sncRNA), i.e. transcripts with length of less than 200 nucleotides that do not serve as template for protein synthesis, have rapidly attracted the interest of researchers in the field of animal reproduction in the last decade, mainly due to their potential use as fertility biomarkers [38]. Bovine spermatozoa are equipped with a wide array of sncRNAs including microRNAs (miRNA) and Piwi-interacting RNAs (piRNA) [39–42]. Several studies have focused on the relation between sperm miRNA profile and bull fertility [40, 43–45]. Although mature sperm are considered transcriptionally silent, their miRNA content shows a dynamic response to stressful procedures, like cryopreservation [34, 46] and induction of capacitation [47]. Indeed, the transcriptome profile of porcine spermatozoa has recently been suggested as an indicator of their freezability and, thus, their ability to tolerate stress related to semen processing [48]. Moreover, it is known that miRNA genes located on the X chromosome are capable of escaping the meiotic sex chromosome inactivation, i.e. the transcriptional silencing of the unsynapsed X- and Y-chromosomal region at the onset of pachynema in mammalian male germ cells [49]. X-linked miRNAs remain active even until the onset of spermiogenesis and serve as post-transcriptional regulators of spermatogenesis at the late meiotic and post-meiotic phases [50]. Despite the increasing evidence about the dynamics of miRNAs in mature sperm and their role in the inactivation/activation cycle of the X and Y chromosome during sperm cell development, their profile in sperm lined up for sex-sorting has not been adequately studied yet.

In the present study, we tested the hypothesis that the miRNA profile of conventional semen is related to the fertility outcome of AI with X-bearing sperm. For this purpose, we assessed the sperm functional status and miRNA profile in conventional AI doses produced from proven sires with diverse fertility after sorting.

## Results

### Descriptive statistics

#### Sperm quality traits

Descriptive statistics (mean value±SD, min and max values) of sperm quality characteristics are presented in Table 1. The samples examined in our study were commercially produced doses that had already passed the post-cryopreservation quality control before being released in the market; thus, not surprisingly the percentage of plasma membrane- and acrosome-intact sperm (PMAI) in both high- (HF) and low-fertility (LF) groups was higher than the commonly applied threshold of 40% (45.96 ± 8.63 and 48.98% ± 8.75% for the LF and HF bulls, respectively). As demonstrated in Table 1, LF bulls had a lower percentage of sperm with high esterase activity, intact plasma membrane, unstained acrosome, low intracellular Ca^2+^ levels and high mitochondrial membrane potential (C_pos_PI_neg_PNA_neg_F_neg_M_pos_; 31.12 ± 6.66 and 35.08% ± 8.19% for the LF and HF group, respectively). The percentage of sperm with high DNA fragmentation index (%DFI) was similar between the two fertility groups (4.01 ± 1.59 and 4.57% ± 2.21% for the LF and HF group, respectively).

**Table 1:** Sperm quality traits in relation to bull fertility group

	LF				HF			
	N	Mean ± SD	Min	Max	n	Mean ± SD	Min	Max
Progressive motility (%)	28	36.21 ± 13.33	9.50	66.60	32	37.82 ± 7.95	19.50	58.60
PMAI sperm (%)	28	45.96 ± 8.63	20.66	58.98	32	48.98 ± 8.75	26.52	63.67
C_pos_PI_neg_PNA_neg_F_neg_M_pos_ sperm (%)	28	31.12 ± 6.66	17.19	45.54	32	35.08 ± 8.19	19.27	55.19
Mean DFI	28	201.91 ± 3.50	198.53	214.29	32	201.02 ± 5.99	179.30	214.94
SD of DFI	28	33.52 ± 11.60	20.53	73.77	32	36.65 ± 9.91	20.95	65.63
%DFI (%)	28	4.01 ± 1.59	2.05	8.65	32	4.57 ± 2.21	2.04	10.14

*LF* low-fertility group, *HF* high-fertility group; n, number of ejaculates, *PMAI* percentage of sperm with intact plasma membrane and unstained acrosome, C_pos_PI_neg_PNA_neg_F_neg_M_pos_ percentage of sperm with high esterase activity, intact plasma membrane, unstained acrosome, low intracellular Ca^2+^ levels and high mitochondrial membrane potential, *DFI* DNA fragmentation index, *SD* standard deviation, *%DFI* percentage of sperm with high DNA fragmentation-index

#### Small RNA sequencing data

**Fig. 1:**
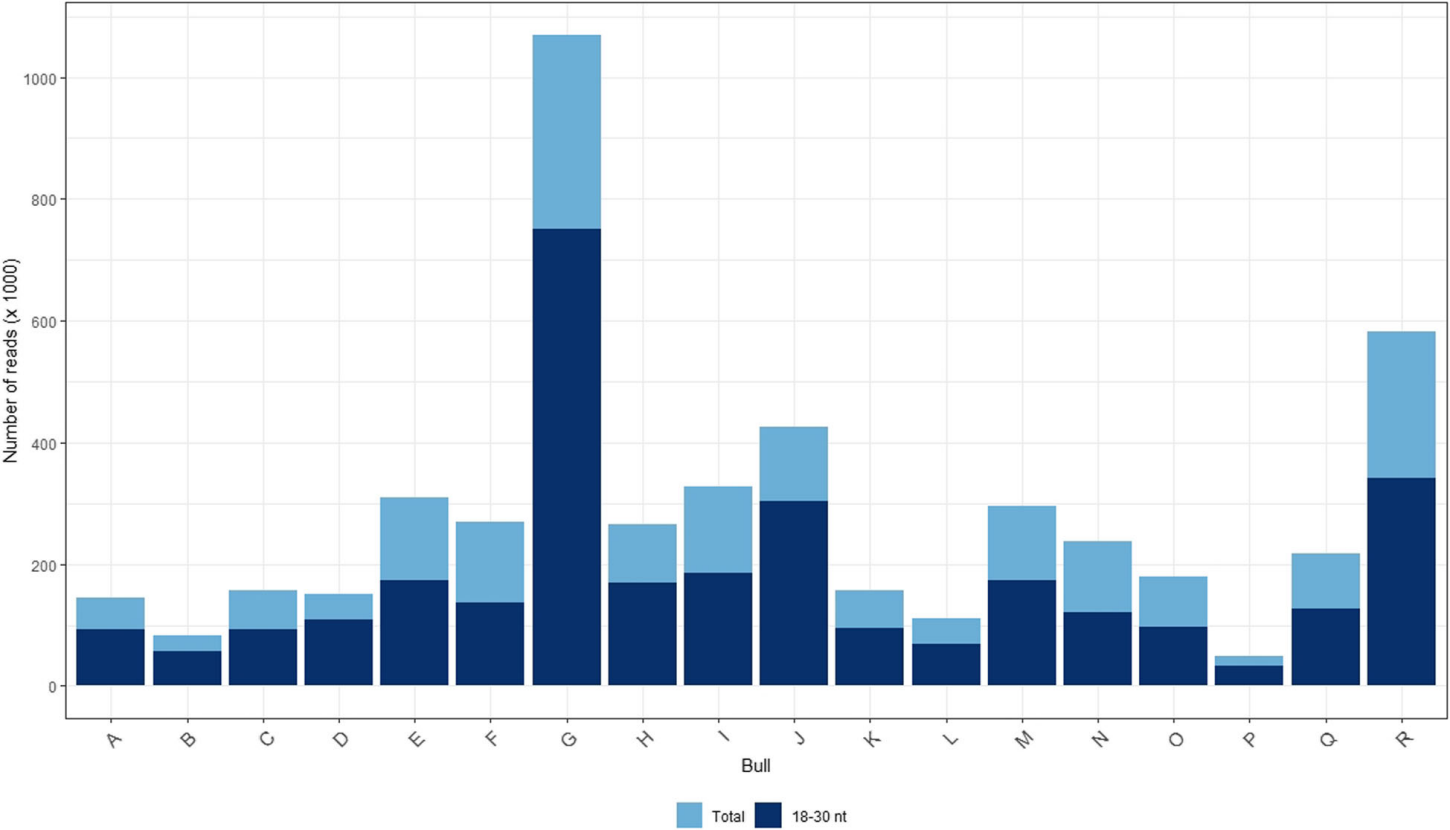
Number of total reads in unsorted sperm samples of 18 bulls. The dark blue bar fraction represents the part of total reads with length of 18–30 nucleotides. Bulls A to I and bulls J to R showed low and high fertility after artificial insemination with X-bearing cryopreserved sperm, respectively

**Fig. 2:**
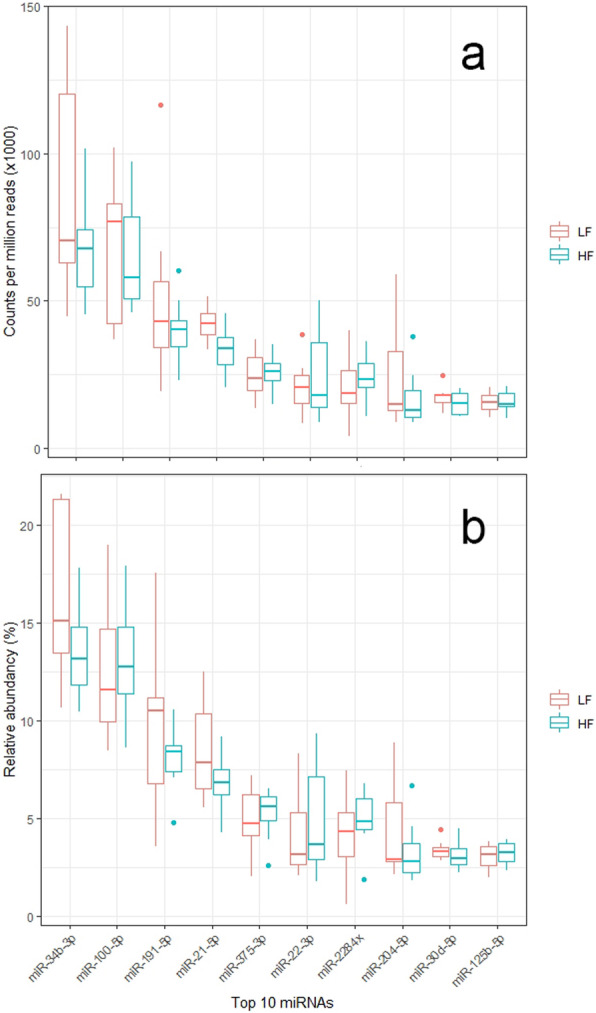
Tukey-style boxplots for the counts per million reads (**a**) and relative abundancy (**b**) of the top 10 miRNAs detected in unsorted sperm samples obtained from low- (LF) and high-fertile (HF) bulls

In total, 48,170 to 1,070,345 reads were identified in each sample (279,763 ± 235,489 reads per sample). More than 50% of the total reads (50.78 to 72.62%) were 18- to 30-nucleotide long (Fig. 1). Across the 18 samples, 4209 unique sequences were identified after filtering of sequences with neglectable read counts. Alignment of unique sequences against bovine and human non-coding and coding sequences revealed 683 sncRNA transcripts in total, with the number of uniquely mapped reads per sample ranging between 5788 and 277,775 reads. Eighty-five miRNAs were identified across the 18 analyzed samples. Counts per million reads (cpm) of the 85 detected miRNAs in the pooled sperm sample of each bull are available in Additional file 1, Table S1. A subset of 55 out of the 85 miRNAs was found in common with miRNAs detected in our previous studies on 30 bovine sperm samples from two cohorts of bulls [43]. The cpm values of the 10 most abundant miRNAs in samples of the LF and HF group are presented in Fig. 2a. MiR-34b-3p and miR-100-5p were the two most highly expressed miRNAs, with their relative abundance reaching approximately 30% in total (Fig. 2b).

#### Correlation between sperm quality traits, miRNA expression levels and fertility data

The Spearman’s rank correlation coefficients (r_s_) describing the relation between miRNA expression levels and sperm quality or fertility traits are presented in Additional file 2. The values of NRR_conv_ were moderately related (0.5 < |r_s_| ≤ 0.7, adjusted *P* < 0.05) to four out of the 85 identified miRNAs (miR-2340, miR-26a, miR-425-5p, miR-151–5p), while NRR_ss_ was significantly correlated with the cpm of nine miRNAs (Additional file 2, Table S2). In particular, the expression levels of miR-9-5p, miR-34c, miR-449a, miR-2483-5p and miR-21–5p were negatively related to NRR_ss_ (− 0.657 ≤ r_s_ ≤ − 0.515, adjusted *P* < 0.05; Additional file 2, Table S2). A moderate positive correlation was detected between NRR_ss_ and the cpm of miR-423-5p, miR-1246, miR-92a and miR-5193-5p (0.521 ≤ r_s_ ≤ 0.693, adjusted P < 0.05; Additional file 2, Table S2). Interestingly, the expression levels of the nine above mentioned miRNAs were not related to the NRR_conv_ or other sperm quality traits, with exception of miR-423-5p and miR-1246 that were correlated to %DFI (r_s_ = − 0.576, adjusted *P* = 0.031) and C_pos_PI_neg_PNA_neg_F_neg_M_pos_ sperm at 0 h (r_s_ = 0.541, adjusted *P* = 0.046), respectively (Additional file 2, Table S2).

### Principal component analysis (PCA)

**Fig. 3:**
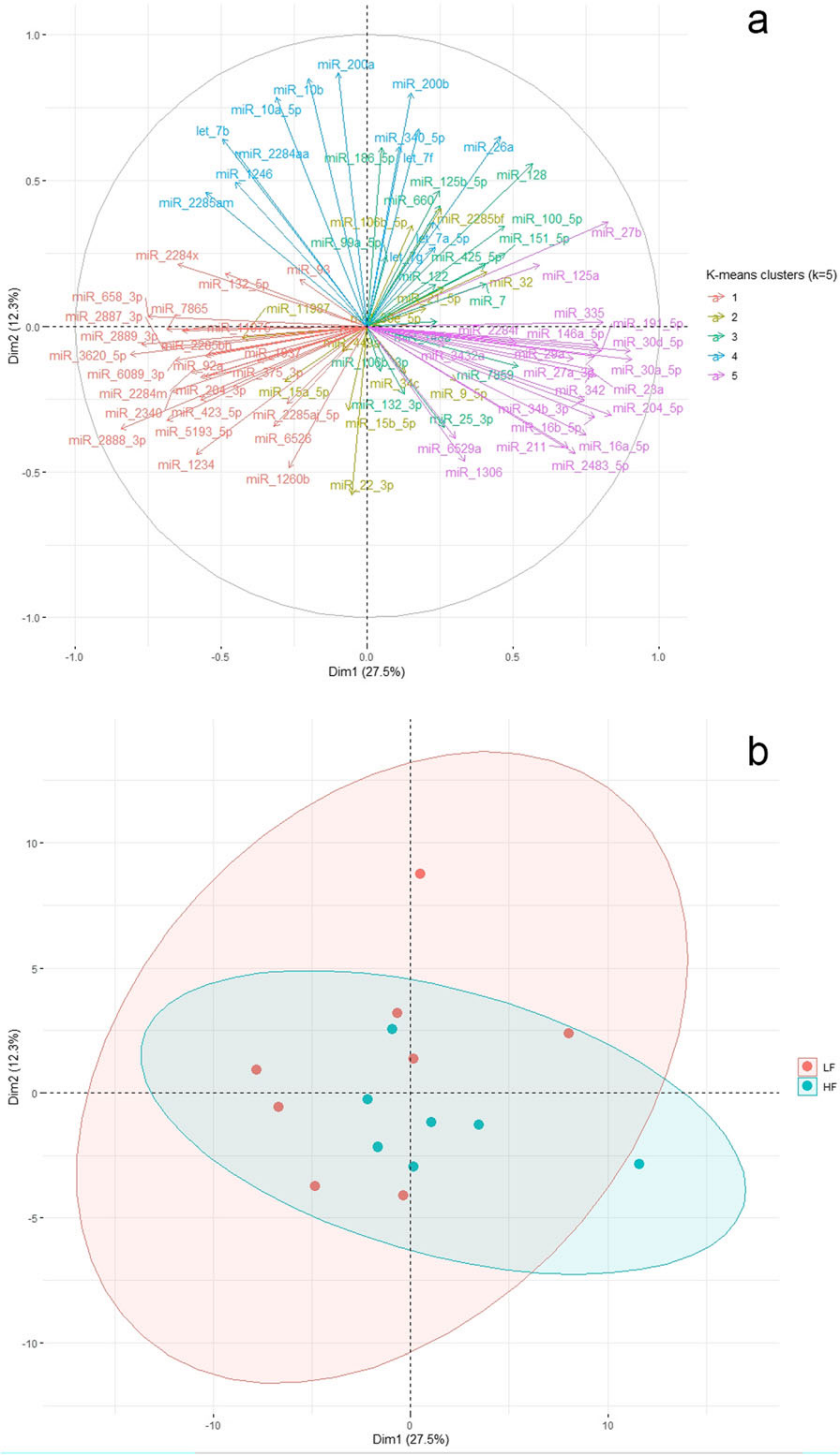
Correlation circle for the 85 identified sperm miRNAs (**a**) and visualization of the sperm samples obtained from low- (LF) and high-fertile (HF) bulls (**b**), plotted against the first two principal components (Dim 1 and 2, respectively)

PCA was performed in an attempt to capture and visualize potential redundancy in the miRNA expression dataset. In total, 14 principal components (PC) were extracted, with the first five of them explaining 68.46% of the dataset’s variance (27.52, 12.35, 10.94, 9.21 and 8.44%, respectively; Additional file 3, Table S3). The coordinates, quality of representation and contribution of the 85 miRNAs to the first five PCs are presented in Additional file 3, Tables S4-S6. The correlations between the first two PCs and the expression levels of the 85 identified miRNAs across the two experimental groups are demonstrated by means of a PCA correlation circle in Fig. 3a. The most characteristic miRNAs for each of the first five PCs, i.e. miRNAs whose expression levels are correlated with single PCs at significance level < 0.05, are presented in Additional file 3, Table S7. The sperm samples obtained from LF and HF bulls could not be distinctly separated when plotting their miRNA expression profile against the first and second PC (Fig. 3b). PCA plots were created for all pairs of the five PCs; however, the results were similar and, thus, not presented here.

### Differential expression analysis

Two out of 85 miRNAs, miR-10a-5p and miR-9-5p, were differentially expressed (DE) between samples of the LF and HF group with a false discovery rate (FDR) of < 10% in both cases. In particular, miR-9-5p was downregulated and miR-10a-5p was upregulated in HF vs. LF sperm samples (− 1.26 and 1.31 log fold change, respectively).

### Robust regression

**Fig. 4:**
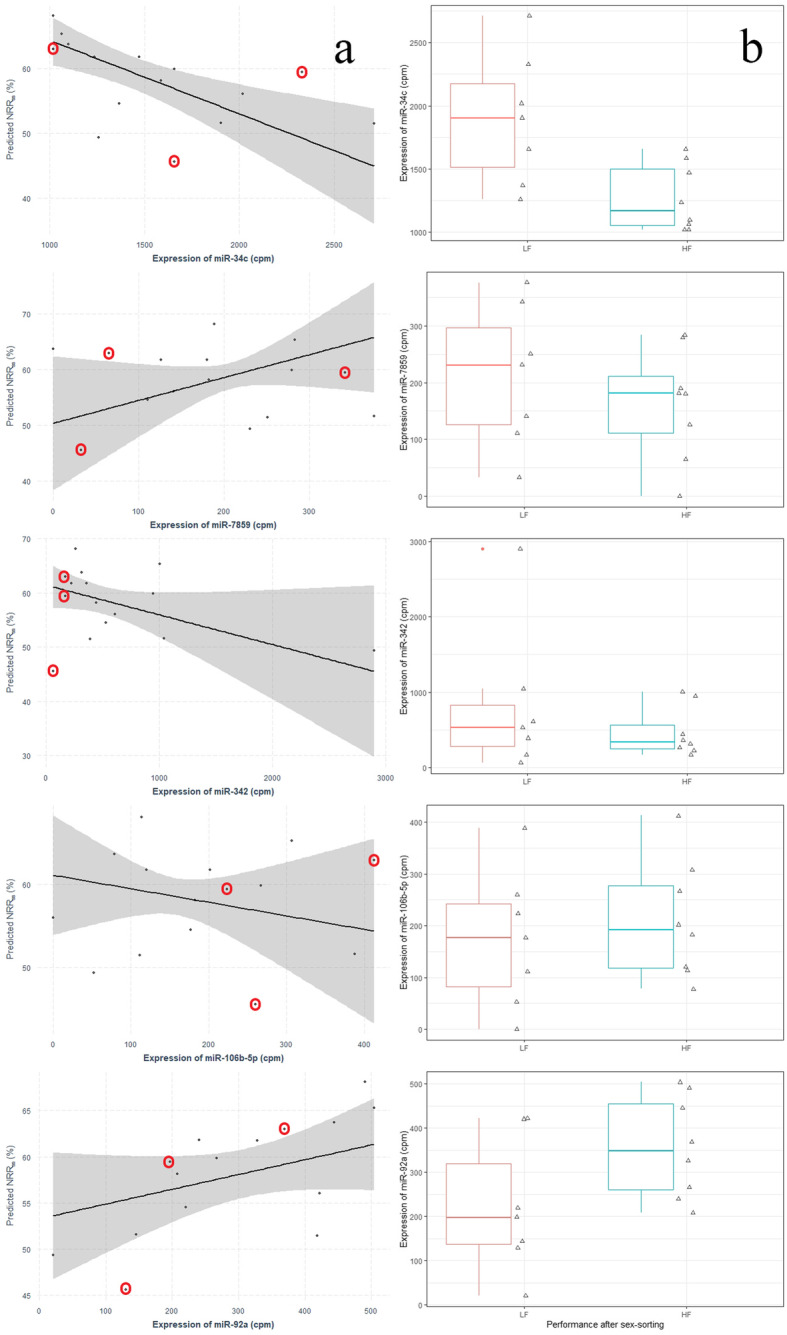
Robust regression lines of five miRNAs predicting the non-return rate for sex-sorted sperm (**a**); Tukey-style boxplots for the expression levels of the five miRNAs in the high-and low-fertility group (**b**). **a**. Robust regression lines with 95% confidence intervals (grey shaded area) for single predictors (predicted NRR_ss_ values plotted against the observed expression levels of single miRNA, when all other predictors are kept constant at their mean value) are presented. Plotted points represent the observed NRR_ss_ values; red circles indicate bulls identified and treated by the robust regression model as outliers in regard to their sperm miRNA profile. NRR_ss_, 56-day non-return rate for sex-sorted sperm; cpm, count per million reads **b**. LF, low-fertility group; HF, high-fertility group

Forward model selection revealed five miRNAs with the highest contribution to the prediction of NRR_ss_: miR-34c, miR-7859, miR-342, miR-106b-5p and miR-92a. Thus, the following robust regression line (M_ss_) was fit:

$$ {NRR}_{ss_i}=a+{b}_1{\left[ miR- 34c\right]}_i+{b}_2{\left[ miR- 7859\right]}_i+{b}_3{\left[ miR- 34 2\right]}_i+{b}_4{\left[ miR- 10 6b- 5p\right]}_i+{b}_5{\left[ miR- 92a\right]}_i+{e}_i $$

where NRR_ss_ is the estimated value of NRR_ss_ for individual i, a the intercept of the regression line, b_1–5_ the coefficients of the respective linear regressors, [miR-x] the expression levels (cpm) of the selected miRNA, and e the additive error term of the model. The variance inflation factor (VIF) of each regressor was computed to evaluate the multicollinearity of expression levels in the subset of the five miRNAs. The regression coefficients b (±SEM) and their respective P and VIF values are shown in Table 2. Values of NRR_ss_ were negatively related to the expression levels of miR-34c (b = − 0.011 ± 0.003, *P* = 0.002) and miR-342 (b = − 0.005 ± 0.001, *P* = 0.022), while the miR-7859 appeared to have a positive effect on NRR_ss_ (b = 0.041 ± 0.014, *P* = 0.009; Table 2). The effect of miR-106b-5p expression levels on the latter was proven not significant (b = − 0.016 ± 0.010, *P* = 0.122; Table 2). Although NRR_ss_ values were positively related to cpm of miR-92a, this trend was not statistically significant (0.016 ± 0.005, *P* = 0.058; Table 2). The NRR_ss_ values predicted with robust regression for each of the five miRNAs (when all other regressors are kept constant at their mean value) are demonstrated in Fig. 4a. The observed expression levels of the five miRNAs in samples of the LF and HF group are presented in Fig. 4b. Three bulls (A, I, K) were identified as outliers based on their sperm miRNA profile (with overall outlying expression of the five regressor miRNAs) and were treated by the robust regression model as so; the above mentioned samples are marked in Fig. 4a.

**Table 2:** Parameters (estimate of coefficients b ± SEM, t statistic and *P* values) of the robust regression line

	Estimate of coefficient b (±SEM)	t statistic	P value	VIF
(*Intercept*)	69.648 ± 6.131	11.360	< 0.001	
miR-34c	−0.011 ± 0.003	−4.461	0.002	2.184
miR-7859	0.041 ± 0.014	3.350	0.009	2.464
miR-342	−0.005 ± 0.001	−2.773	0.022	2.513
miR-106b-5p	−0.016 ± 0.010	−1.707	0.122	1.683
miR-92a	0.016 ± 0.005	2.171	0.058	1.623

*SEM* standard error of the mean, *VIF* variance inflation factor

**Fig. 5:**
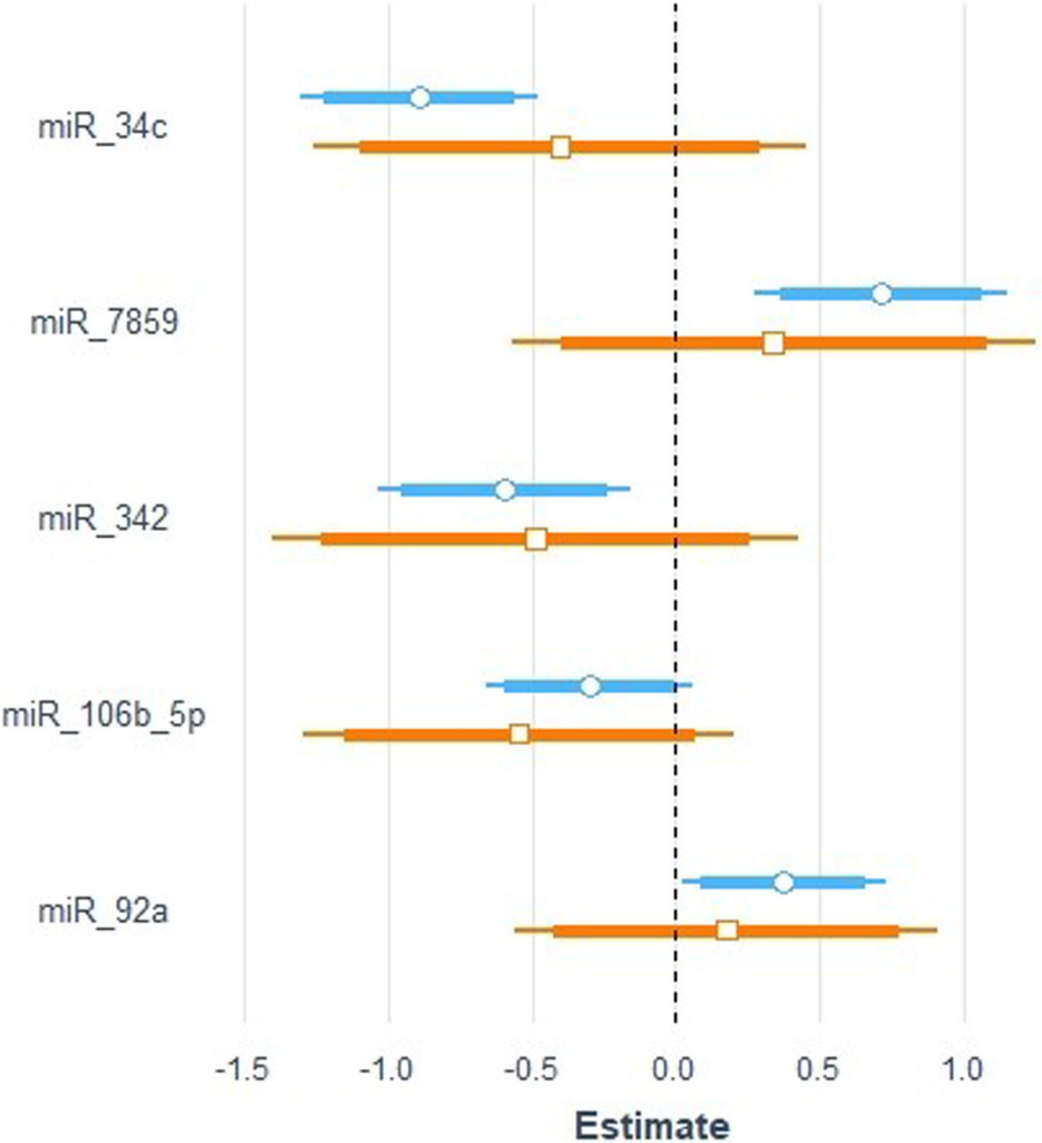
Standardized b coefficients for two robust regression models describing the relation between the expression levels of five sperm miRNAs and the 56-day non-return rate of 15 bulls recorded after artificial insemination with unsorted (orange) and sex-sorted (blue) cryopreserved sperm, respectively. Transparent points represent the mean of the standardized coefficient b, the horizontal line the 95% confidence intervals and the thicker part of the line the 90% confidence intervals of the estimated coefficients

In a following step, we tried to explore whether the five selected miRNAs made an actual contribution to the variance of the outcome variable NRR_ss_ or indirectly affected the sire’s performance after sex-sorting through its overall fertility status. Therefore, NRR_conv_ was modeled as a function of the five miRNAs (model M_conv_). The standardized b coefficients and the confidence intervals of models M_ss_ and M_conv_ are graphically demonstrated in Fig. 5. The regression coefficients describing the relation of the five selected miRNAs to NRR_conv_ were closer to zero, while their 95% confidence intervals crossed the vertical zero-threshold line, apparently indicating non-significance of the M_conv_ model parameters. Therefore, it was confirmed that the five selected miRNAs had a direct effect on the fertilizing potential of sex-sorted sperm and did not affect its performance through the general fertility status of the bull.

### Functional annotation of miRNA predicted targets

#### DE miRNAs (miR-9-5p and miR-10a-5p)

**Fig. 6:**
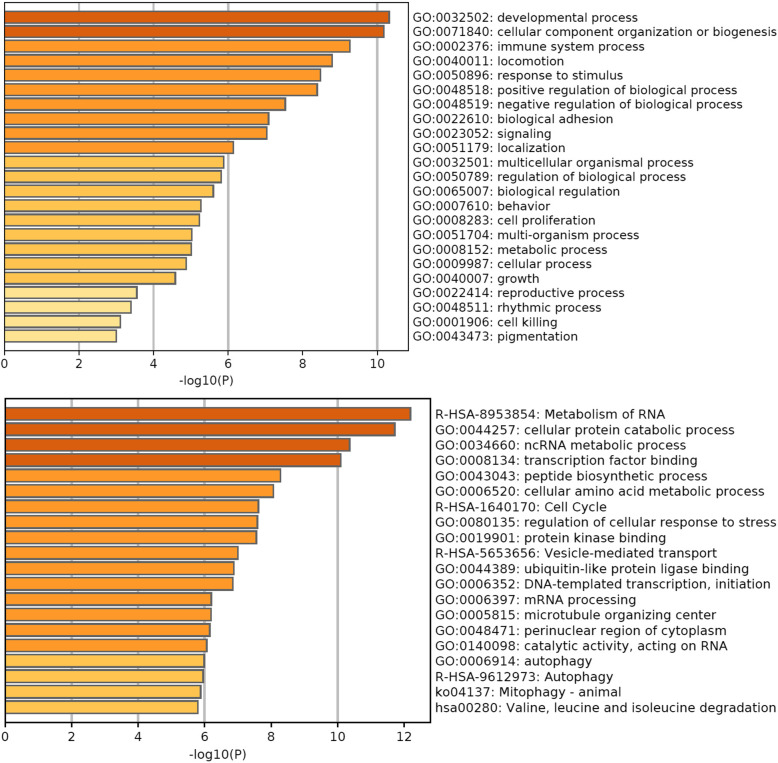
The top 20 gene ontology enriched clusters (biological processes) for the predicted targets of the two differentially expressed miRNAs (miR-9-5p, miR-10a-5p; top panel), and four out of five robust regression predictors (miR-34c, miR-342, miR-106b-5p, miR-92a; bottom panel), colored for P value. log10(P), P value in log base 10

In total, 442 potential target genes were detected for the two DE miRNAs (FDR<5%; Additional file 4, Table S8). The most significant enriched GO terms were related to developmental process, cellular component organization or biogenesis, immune system process, locomotion and response to stimulus (Fig. 6). Other interesting GO terms were associated to regulation of biological process, localization, cell proliferation, biological adhesion and reproductive process (Fig. 6). The most representative enriched GO terms of the top 20 clusters (one term per cluster) along with their P and multi-test adjusted *P* values (q) are presented in Table 3. The complete list of GO terms with a score ≥ 1.3 is available in Additional file 4, Table S9.

**Table 3:** Top 20 clusters with their representative enriched terms (one per cluster)

GO	Category	Description	Count	%	Log10(P)	Log10(q)
GO:0084708	GO Biological Processes	astrocyte differentiation	14	3.17	−10.32	− 6.01
GO:0043062	GO Biological Processes	extracellular structure organization	30	6.79	− 10.18	− 6.01
GO:0007420	GO Biological Processes	brain development	39	8.82	−9.85	−5.86
GO:0001568	GO Biological Processes	blood vessel development	39	8.82	−9.32	−5.57
GO:1903706	GO Biological Processes	regulation of hemopoiesis	30	6.79	−9.26	−5.57
GO:0007423	GO Biological Processes	sensory organ development	32	7.24	−8.98	−5.51
GO:0040017	GO Biological Processes	positive regulation of locomotion	33	7.47	−8.79	−5.37
GO:0048732	GO Biological Processes	gland development	27	6.11	−8.52	−5.13
GO:0009611	GO Biological Processes	response wounding	35	7.92	−8.48	−5.13
GO:0005912	GO Biological Processes	adherens junction	29	6.56	−7.65	−4.48
GO:0010942	GO Biological Processes	positive regulation of cell death	34	7.69	−7.35	−4.26
GO:0030424	GO Biological Processes	axon	31	7.01	−7.31	−4.24
GO:0061061	GO Biological Processes	muscle structure development	32	7.24	−7.26	−4.22
GO:0031589	GO Biological Processes	cell-substrate adhesion	22	4.98	−7.08	−4.14
GO:1905114	GO Biological Processes	cell surface receptor signaling pathway involved in cell-cell signaling	30	6.79	−7.03	−4.11
GO:0043583	GO Biological Processes	ear development	17	3.85	−6.93	−4.02
GO:0001501	GO Biological Processes	skeletal system development	26	5.88	−6.58	−3.76
GO:0045165	GO Biological Processes	cell fate commitment	18	4.07	−6.31	−3.53
GO:0043009	GO Biological Processes	chordate embryonic development	29	6.56	−6.23	−3.49
GO:0045860	GO Biological Processes	positive regulation of protein kinase activity	26	5.88	−6.21	−3.48

Count, number of genes in the user-provided lists with membership in the given ontology term; %, percentage of all user-provided genes in the given ontology term (only input genes with at least one ontology term annotation are included in the calculation); Log10(P), *P* value in log base 10, Log10(q), multi-test adjusted *P* value in log base 10

To further capture the relationships between the enriched GO terms, a subset of them was selected and rendered as a network plot, where terms with a similarity > 0.3 were connected by edges. We selected the terms with the best P values from each of the 20 clusters, with the constraint that there were no more than 15 terms per cluster and no more than 250 terms in total. For network visualization, each node represented an enriched term and was colored by its cluster ID (Additional file 5, Figure A) and by its *P* value (Additional file 5, Figure B).

#### Robust regression predictor miRNAs (miR-34c, miR-7859, miR-342, miR-106b-5p and miR-92a)

Six hundred and eight potential target genes (Additional file 4, Table S10) of four out of five miRNAs used for robust regression (miR-34c, miR-342, miR-106b-5p, miR-92a) were identified (FDR < 5%); miR-7859 was not included in existing databases for human species. The top enriched GO terms were associated to metabolic processes, transcription factor binding, response to stimulus, cell cycle and protein kinase binding. GO terms linked to vesicle-mediated transport, ubiquitin-like protein ligase binding, valine, leucine and isoleucine degradation, autophagy and mitophagy were also identified. The complete list of GO terms with a score ≥ 1.3 is available in Additional file 4, Table S11.

## Discussion

The use of sex-sorted sperm in bovine assisted reproduction is constantly expanding; however, it is frequently observed that bulls with high fertility after AI with conventional sperm may not perform optimally when sex-sorted doses are used for inseminating either cows or heifers. In the present study, we explored the relation between the miRNA profile of conventionally produced semen doses and the fertility status of the bull after AI with sex-sorted sperm.

Our analysis revealed a wide variety of miRNAs in bovine sperm, with miR-34b-3p and miR-100-5p comprising approximately 30% of the analyzed sperm miRNAome. The most abundant miRNA, miR-34b-3p, was present in both HF and LF bulls with a relative abundancy of approximately 15%. Similar values have been previously reported for bovine sperm in other studies [51, 52]. It has been shown that transcripts of the miR-34b/c cluster are preferentially expressed in the testis and play a crucial role in sperm chromatin condensation in the stage of pachytene spermatocytes and round spermatids [53]. Based on the outcome of robust regression analysis, miR-34c but not miR-34b-3p was highlighted as a deciding predictor of NRR_ss_. Even though the two miRNAs are co-transcribed from a common cluster (miR-34b/c) on bovine chromosome 15, their expression and function can largely vary within the same cell type [51]. This could probably explain the relation of miR-34c but not of miR-34b-3p with NRR_ss_ in our study.

Our results suggested a negative correlation between miR-34c expression levels and the fertility of sperm after sex-sorting. MiR-34c has been detected in sperm of several species, including the equine [54], porcine [55], murine [56] and human [57–59], and is considered as one of the most abundant miRNAs in bull sperm and male germ cells [42, 51, 60]. MiR-34c profoundly plays a role in the growth, differentiation and apoptosis of the male germ cell line through regulation of the transforming growth factor beta and the notch signaling pathways [61]. Individuals not able to express the seed sequence of miR-34c (i.e. lacking miR-34c and miR-449 simultaneously) have lower sperm concentration and impaired sperm kinetics [53]. In the same direction, Capra et al. (2017) reported elevated miR-34c expression levels in the high-motile fraction of cryopreserved bovine sperm selected by means of Percoll density gradient centrifugation [52]. In our study, miR-34c cpm were related neither to the functional traits of conventional sperm nor to NRR_conv_. This could be apparently attributed to the low between-bull variability of the latter. However, it is not easy to explain why sperm with lower miR-34c expression performs better after sex-sorting, as indicated by our analysis. Recent studies have highlighted the importance of an intense co-expression and target network of miRNAs involved in the manifestation of complex phenotypic traits including bovine fertility [42, 62]. In the light of this information, one could speculate that pre-sorting elevated miR-34c expression indirectly affects the fertilizing ability of sperm after sorting, possibly through alterations in the expression or function of interrelated miRNAs. Interestingly, the profile of sperm miR-34c is susceptible to semen processing and particularly cryopreservation [46]. Future split-ejaculate experiments focusing on the effect of sex-sorting on sperm miRNA profile could elucidate the role of miR-34c in the fertility of sex-sorted sperm.

As suggested by our results, miR-342 was also included in the predictors of NRR_ss_. This miRNA has been previously identified in various bovine tissues [63, 64] as well as in the testes and sperm of other mammalian species [65–67]. MiR-342 belongs to the long list of miRNAs whose relative abundance substantially changes during epididymal maturation [56], a process that renders sperm their ability to capacitate once deposited in the female genital tract. Furthermore, miR-342 can block the lipogenesis-cholesterogenesis pathway [68] which is directly linked to the capacitation of mature sperm [69, 70]. Premature capacitation-like changes of sperm structure and function during sorting are counted among the main causes for the reduced longevity of sex-sorted sperm [71–73]. In our study, miR-342 expression levels in conventional sperm samples were negatively correlated with NRR_ss_ but not with NRR_conv_ or the inducibility of acrosome reaction of conventional sperm (flow cytometrically assessed after challenging sperm with calcium ionophore A23187 and dual PI/PNA staining; data not shown). Thus, any potential effect of miR-342 expression on the fertilizing ability of X-bearing sperm was apparently not linked to the capacitation status of spermatozoa before sorting.

Remarkably, our analysis and particularly forward model selection pointed out miR-7859, a miRNA that had not been previously described in bovine germ cells or sperm, as one of the five predictors of NRR_ss_. MiR-7859 has been previously documented as part of the microRNAome of the bovine [74] and caprine mammary gland [75], but information about its functional role are rather scarce. Based on our results, the expression levels of miR-7859 appeared to have a positive predictive value for NRR_ss_; however, miR-7859 transcripts were on average more abundant in LF than in HF bulls. At this point, one should note that miR-7859 and miR-106b-5p, another predictor of NRR_ss_ values in our robust regression model, were listed among the five least abundant miRNAs in the examined samples. It is most likely that the low expression levels of miR-7859 in sperm samples of both groups along with the relatively small sample size of our experiment are responsible for these inconsistent observations.

Regarding miR-92a, its expression levels were distinctly lower in conventional sperm produced by bulls with sub-optimal performance after sex-sorting. However, the positive predictive value of miR-92a for NRR_ss_ was proven marginally not significant. Even when the weakly expressed miR-7859 and miR-106b-5p were excluded from robust regression analysis, the effect of miR-92a on NRR_ss_ was still not significant (data not shown). MiR-92a and miR-106b-5p are members of the multifunctional paralog gene clusters miR-17-92 and miR-106b-25, respectively, and are considered essential for the undisrupted progress of spermatogenesis [76–78]. MiR-17-92- and miR-106b-25-deficient mice display extended loss of spermatogonia and spermatogonial stem cells, and, consequently, testicular atrophy and reduced sperm production [76, 77]. The small number of sperm that reach the epididymis of miR-17-92-mutant animals exhibit normal morphology and motility characteristics [77], which implies that miR-92a might have an effect on male fertility but in a manner not directly linked to conventional sperm quality characteristics. Interestingly, Tong et al. (2012) showed that the expression of miR-106b-25 cluster increases dramatically in the germ cells of miR-17-92 knockout male mice, while sperm quality of the latter remains unchanged [76]. This observation urged the authors to suggest that the members of the two paralog clusters miR-17-92 and miR-106b-25 function in a redundant way in sperm cells. Apparently, impairment of the function of either of the two miRNA clusters can be compensated by the other, so that no phenotypic alterations are observed in the produced sperm [76]. In the light of this information, one could attribute the inconsistent effect of miR-92a and miR-106b-5p on NRR_ss_ to the well documented functional interrelation of the two miRNAs and potential collinearity issues in the robust regression model.

When we compared the miRNA profiles between sperm samples of the two artificially created groups LF and HF, two out of the 85 sperm miRNAs were found to be differentially expressed. MiR-9-5p and miR-10a-5p were down- and upregulated in the HF and LF sperm, respectively. Transcripts of miR-10a have previously been detected in mature bovine sperm [52, 60], while miR-9-5p has been frequently reported in porcine sperm [34, 46, 79]. It has been shown that both miRNAs are upregulated in porcine sperm after cryopreservation [34, 46]; however, there is no information regarding the effect of sex-sorting on miR-10a-5p and miR-9-5p profiles. In the bull, miR-10a-5p is upregulated in sperm with low motility [52]. Furthermore, overexpression of miR-10a-5p in late spermatogenesis can adversely affect the DNA repair capacity of murine spermatocytes leading to severe testicular atrophy and infertility in adulthood [80]. Based on the literature described above, the upregulation of miR-10a-5p is linked to impaired sperm quality, which makes it difficult to explain the overexpression of miR-10a-5p in sperm samples obtained from HF bulls in our study.

The production of cryopreserved sex-sorted sperm comes with inherent stress, like the elevated sorting pressure and the subsequent freezing steps, which substantially impairs sperm quality. Changes of sperm morphometry [81] and function [82] are well documented consequences of mechanical stress during sorting. Although there is some indication for the importance of miRNAs for sperm freezability [34], their role in the resistance of sperm against mechanical stimuli has not been studied yet. Several miRNAs mediate metabolic changes induced by mechanical stress in other types of cells [83–85], for example miR-92a, miR-34c and miR-21–5p that were correlated with NRR_ss_ values in our study. Nevertheless, the contribution of miRNAs, if any, to the response of bovine sperm to sorting stress is still to be clarified.

In the present study, we intentionally selected bulls with uniform NRR_conv_ but with diverse fertility after AI with sex-sorted sperm, in order to avoid the masked effect of a sire’s general fertility status on his performance after sex-sorting. The examined semen doses had successfully passed the post-thaw quality control before being used for commercial AI in the field, and laboratory assessed traits of conventional sperm showed relatively low between-bull variability. Given the limited variance of NRR_conv_ and sperm quality characteristics, it is not surprising that only few miRNAs were related to the above-mentioned parameters. In contrast to differential expression analysis, robust regression enabled the analysis of sperm miRNAome in relation to continuous fertility and sperm variables without losing within-group phenotypic variation; however, limitations in the interpretation of the results due to the small sample size of our study must be considered.

## Conclusions

In conclusion, we were able to detect 85 miRNAs in conventional cryopreserved semen doses collected from proven sires that were in parallel used for the production of sex-sorted sperm. Our analysis revealed that NRR_ss_ values were related to the expression levels of five out of the 85 identified miRNAs (miR-34c, miR-342, miR-7859, miR-106b-5p and miR-92a) but not to the post-thaw quality characteristics of conventional sperm. This finding raises questions regarding the mechanisms by which these miRNAs could affect the performance of sperm after sex-sorting. Whether they are responsible for subtle alterations of spermatozoal phenotypes that are only manifested after sex-sorting or they specifically interfere with the performance of X-bearing sperm, remains to be clarified. Studies with higher numbers of samples and split-ejaculate experiments focusing on miRNA profiles of sex-sorted compared to conventional sperm are apparently necessary to validate the suitability of miRNAs as markers for the fertilizing potential of sex-sorted sperm.

## Methods

### Biological material

#### Bull and sperm sample selection

For this study, 18 bulls (*Bos taurus taurus*) were selected from a pool of 50 sires housed in a single AI station under uniform management and feeding conditions. All bulls were in parallel used for the production of conventional and sex-sorted (X-bearing sperm at 90% purity) cryopreserved sperm doses. Fertility of sires after AI with conventional and sex-sorted semen was systematically recorded in form of an annual % 56-day NRR after a minimum of 500 first services (NRR_conv_ and NRR_ss_, respectively). For each bull the relative change of the NRR (Δ_NRR_) after AI with conventional and sex-sorted semen was computed according to the following formula:

$$ {\varDelta}_{NRR}\left(\%\right)=100\times \left[\left({NRR}_{conv}-{NRR}_{ss}\right)/{NRR}_{conv}\right] $$

Eighteen bulls with Δ_NRR_ values lying at the extremes of the Δ_NRR_ distribution of the 50 sires, i.e. outside the range of mean Δ_NRR_ ± SD, were selected and assigned in two equal groups: nine bulls (five Holstein-Friesian, one Red Holstein, two Swiss Fleckvieh and one Simmental bull) with low fertility (LF; n_LF_ = 9), and nine bulls (three Holstein-Friesian, two Red Holstein, three Brown Swiss and one Limousin bull) with high fertility (HF; n_HF_ = 9) after sex-sorting. The mean values±SD of fertility data (number of first services with conventional and sex-sorted sperm, NRR_conv_, NRR_ss_, Δ_NRR_) in relation to fertility group are presented in Table 4. Bulls of both groups showed similar NRR_conv_ values (69.57 ± 3.33 and 68.61% ± 2.09% for LF and HF bulls, respectively); however, the NRR values of LF bulls were reduced by 22.33% after AI with sex-sorted sperm against a reduction of only 8.63% observed for the HF bulls (Table 4).

**Table 4:** Fertility data of 18 bulls in relation to their fertility group

	LF				HF				Total			
	n	Mean ± SD	Min	Max	n	Mean ± SD	Min	Max	n	Mean ± SD	Min	Max
First AI with conventional sperm	9	7232 ± 4466	1931	13,656	9	11,549 ± 16,817	613	57,705	18	9391 ± 12,491	613	57,705
First AI with sex-sorted sperm	9	1208 ± 357	741	1836	9	2674 ± 2805	520	9863	18	1941 ± 2130	520	9863
NRR_conv_ (%)	9	69.57 ± 3.33	64.11	73.85	9	68.61 ± 2.09	64.97	72.58	18	69.09 ± 2.82	64.11	73.85
NRR_ss_ (%)	9	54.10 ± 4.70	45.58	59.81	9	62.69 ± 2.74	58.13	68.10	18	58.40 ± 5.77	45.58	68.10
Δ_NRR_ (%)	9	22.33 ± 4.01	31.44	18.84	9	8.63 ± 1.71	10.53	5.25	18	15.48 ± 7.50	31.44	5.25

*LF* low-fertility group, *HF* high-fertility group, *n* number of examined bulls, *AI* artificial insemination, *NRR*_*conv*_ 56-day non-return rate for conventional sperm, *NRR*_*ss*_ 56-day non-return rate for sex-sorted sperm, Δ_NRR_ relative change of NRR_ss_ against NRR_conv_

#### Semen collection and processing

The cryopreserved sperm samples examined in this study originated from the regular semen collection schedule of the AI center. Semen was collected by using a pre-warmed (38 °C) artificial vagina after the bulls mounted on a dummy bull or cow. Ejaculates were evaluated immediately after ejaculation in terms of ejaculate volume, sperm concentration, progressive motility and morphology using a phase contrast microscopy with 100× magnification. Only ejaculates fulfilling the criteria of volume ≥ 1 mL, sperm concentration ≥ 300 × 10^6^ sperm/mL and progressive motility ≥70% were further processed and cryopreserved.

Semen was extended with a Triladyl®-egg yolk extender [250 g Triladyl® (Minitube GmbH, Tiefenbach, Germany), 750 mL distilled water, 250 mL egg yolk] to a final concentration of 71 × 10^6^ sperm/mL and packaged in 0.25 mL French straws (IMV Technologies; L’Aigle, France) using a fully automatic straw filling and sealing machine (IS4, IMV Technologies, Aîgle, France). Semen was then cooled to 4 °C for 24 h before freezing by means of a computer-assisted freezing chamber (Digitcool 5300 3 T, IMV Technologies, Aîgle, France) with a temperature decrease rate of 5 °C/min to − 10 °C, then 40 °C/min to − 110 °C and finally 20 °C/min to − 140 °C. Right after, straws were transferred and stored in liquid nitrogen (− 196 °C).

In total, 72 unsorted and cryopreserved ejaculates (four conventional semen batches per bull) were used as input for laboratory semen examination and analysis of their small non-coding RNA profile.

### Laboratory sperm analysis

#### Preparation of semen prior to analysis

Four straws from each batch were thawed in a water bath (38 °C, 30 s) and pooled in a pre-warmed (38 °C) 1.5-mL laboratory tube. Pooled samples were further assessed with computer-assisted sperm analysis and flow cytometry immediately after thawing (0 h) at 38 °C. Materials and buffers or staining solutions contacting with sperm were pre-warmed at 38 °C.

#### Computer-assisted sperm analysis (CASA)

Sperm concentration, motility and kinematics were assessed using an IVOS II CASA system driven by the software version 1.10.1 *(*Hamilton Thorne Inc., Beverly, U.S.A.). A pre-warmed (38 °C) 20 μm-deep 4-chamber Leja slide (IMV Technologies; L’Aigle, France) was filled with 6 μL of semen, and a minimum of 1000 cells were analyzed in at least eight randomly selected fields with 30 frames acquired per field at a frame rate of 60 Hz. Sperm with straightness ≥70% and average path velocity ≥ 50 μm/s were considered progressively motile. In each sample the percentage of progressively motile sperm (progressive motility) was recorded.

#### Flow cytometric analysis of sperm

##### Chemicals and reagents

Chemicals used for the preparation of Tyrode’s solution, Tris-NaCl-EDTA (TNE) buffer (0.01 M Tris, 0.15 M NaCl, 1 mM EDTA, pH 7.4), acid detergent solution (0.15 M NaCl, 0.08 N HCl, 0.1% Triton-X 100, pH 1.2), acridine orange (AO) staining buffer (0.2 M Na_2_HPO_4_, 1 mM EDTA, 0.15 M NaCl, 0.1 M citric acid, pH 6.0) as well as propidium iodide (PI) were purchased from Sigma-Aldrich Co. (Buchs, Switzerland). The fluorochromes CELLTRACE Calcein Violet AM, Fluo-4 AM and 1,1′,3,3,3′,3′-Hexamethylindodicarbocyanine iodide [MITOPROBE DiIC_1_(5)] were obtained from Thermo Fisher Scientific Inc. (Waltham, U.S.A.), while AO and the phycoerythrin-conjugated agglutinin of *Arachis hypogea* (PE-PNA) were purchased from Polysciences Europe GmbH (Eppelheim, Germany) and GeneTex Inc. (Irvine, U.S.A.), respectively.

The purchased fluorescent probes were diluted and used for sperm staining in form of working solutions as described here: 25 μg calcein violet AM/52 μL dimethyl sulfoxide (DMSO; 1.21 μM); 10 mg PI/5 mL double-distilled water (2.99 mM PI); 1 mg/mL PE-PNA; 50 μg Fluo-4 AM/225 μL DMSO (2 μM); 10 μM DiIC_1_(5) in DMSO (0.015 μM).

##### Sperm chromatin structure assay™ (SCSA™)^1^

The SCSA was performed to evaluate the susceptibility of sperm to acid-induced DNA fragmentation at 0 h, using a COULTER EPICS XL flow cytometer driven by EXPO32 ADC XL 4 COLOR software (Beckman Coulter Inc., Krefeld, Germany). In short, 400 μL of acid detergent solution were added to 200 μL of semen previously diluted with TNE buffer to a final concentration of 1 to 2 × 10^6^ sperm/mL. Following the mixing of the sample for 30 s, 1.2 mL of AO staining solution (6.0 μg AO/mL AO staining buffer) were added and stained samples were assessed by flow cytometry after exactly 3 min. Cells were excited by a 488-nm argon laser and the emitted green and red fluorescence was captured by means of a 525/20 and a 620/15 band-pass (BP) filter, respectively. In total, 10,000 cells were analyzed for each sample at a flow rate of 200 cells/sec. Flow cytometric data analysis was performed using the 4.07.0005 version of FCS EXPRESS 4 Flow Cytometry Research Edition software (De Novo Software, Glendale, U.S.A.). The mean value and SD of the DNA fragmentation index (DFI) as well as the percentage of cells with high DFI (%DFI) were computed as previously described by Evenson and Jost (2001) [87].

##### Multicolor flow cytometric assay^2^

The multicolor assay was performed using the CytoFLEX Flow Cytometer V5-B5-R3 operated by the CytExpert Software for CytoFLEX version 2.1 (Beckman Coulter Inc., Nyon, Switzerland), as previously described by Bucher et al. (2019) [88]. The flow cytometer included five channels from the violet (405 nm) laser, five channels from the blue (488 nm) laser, and three channels from the red (638 nm) laser. The violet, blue, and red solid-state diode lasers operated with a power of 80 mW, 50 mW, and 50 mW, respectively. Flow rate was set to 60 μL/min and 500 to 1000 events/sec; for each sample 10,000 cells were analyzed. A fluorescent panel consisting of calcein violet AM, PI, PE-PNA, Fluo-4 AM and DiIC_1_(5) was employed for the simultaneous evaluation of intracellular esterase activity, plasma membrane integrity, acrosomal status, intracellular Ca^2+^ levels, and mitochondrial membrane potential of sperm, respectively. The laser and band-pass (BP) filters used for the excitation and detection of emission signal of each fluorophore, respectively, are presented in Additional file 7.

For the examination of each sperm sample with the multicolor assay, sperm was diluted to a concentration of 1.2 × 10^6^ sperm/mL with Tyrode’s solution at a final volume of 244.75 μL in a 250-μL reaction well of a 96-well plate. Just prior to the performance of the assay, the fluorescent probes were combined in a master mix solution consisting of 0.375 μL calcein violet AM, 1.5 μL PI, 0.5 μL PE-PNA, 2.5 μL Fluo-4 AM, and 0.375 μL DiIC_1_(5) per reaction well. Thus, 5.25 μL of master mix were added to each reaction well. After 15 min of incubation at 38 °C, sperm were analyzed by flow cytometry.

The % size of the sperm sub-population simultaneously exhibiting the following features was quantified: high esterase activity, intact plasma membrane, PE-PNA-unstained acrosome, low [Ca^2+^]_i_ and high mitochondrial membrane potential. Values of PMAI were also determined.

### Analysis of sperm small non-coding RNA profile

#### Sperm RNA extraction

##### Sperm homogenization

Total RNA was extracted from cryopreserved bovine sperm using a modified heated TRIzol® Reagent-based protocol (TRIzol® Reagent, Invitrogen, ThermoFisher Scientific, Waltham MA, U.S.A.). Using the CASA system, the sperm concentration of single 0.25-ml straws was assessed post-thaw; straws of the LF and HF group had a mean concentration of 60.4 × 10^6^ and 49.9 × 10^6^ sperm/mL, respectively; thus, 50–60 × 10^6^ cells were used as input for total RNA extraction. Sperm samples of each bull (four cryopreserved ejaculates per bull, one straw per ejaculate) were thawed on ice and pooled immediately after thawing; the sperm pellet was separated from semen extender after centrifugation at 5000×g for 5 min (4 °C). Homogenization of harvested sperm was performed according to the procedure previously described by Rauber (2008) [89] in order to control potential somatic cell contamination. In short, the sperm pellet was re-suspended with 1 mL of ice-cold hypotonic somatic cell lysis buffer (10 mM Tris HCl, 50 mM KCl, 2.5 MgCl_2_, 4 mM DTT, 0.05% w/v SDS, 0.5 v/v Triton-X 100; pH 7.4), incubated on ice for 10 min and thereafter centrifuged at 5000×g for 5 min (4 °C). After discarding the supernatant, the harvested pellet was washed with 1 mL of ice-cold 1× PBS (Invitrogen, ThermoFisher Scientific, Vilnius, LT) and centrifuged at 5000 for 5 min (4 °C). Then, the re-harvested pellet was suspended with 1.5 mL of ice-cold TRIzol® Reagent (1.5 mL TRIzol®/1.2 × 10^6^ sperm). In order to disrupt the plasma membrane of sperm, the TRIzol®-sperm suspension was passed four times through a 25G needle and vigorously mixed for 30 s.

##### Total RNA extraction

Samples were centrifuged at 12,000×g for 10 min (4 °C). The harvested pellet consisted of insoluble material, such as membranes, polysaccharides and high molecular weight DNA [89] and was, therefore, discarded; RNA was contained in the supernatant, which was transferred in a new tube, and heated at 65 °C for 10 min while being mixed at 600 rpm. Right after, 200 μL chloroform/mL TRIzol® were added and samples were vigorously mixed for 30 s. Following a 3-min incubation at room temperature, samples were centrifuged (12,000×g, 4 °C, 15 min) and 500 μL of the aqueous phase containing the spermatozoal total RNA were separated and transferred to a new tube that included 500 μL of 100% Isopropanol/mL TRIzol®. Total RNA was allowed to precipitate for 45 min at room temperature after the addition of 10 μg RNase-free glycogen/mL TRIzol®. The RNA pellet was harvested after centrifugation at maximum relative centrifugal force (rcf) for 30 min (4 °C), twice washed with 1 mL 75% Ethanol/mL TRIzol® (centrifugation between the washing steps at maximum rcf for 5 min, 4 °C) and re-suspended with 10 μL pyrogen free DEPC-treated water (Invitrogen, Carlsbad, CA). Then, the pellet was dissolved after 10 min incubation on ice.

##### DNase-treatment

Collected spermatozoal RNA was treated with RNase-free recombinant DNase I (Invitrogen, ThermoFisher Scientific, Waltham MA, U.S.A.) according to the protocol provided by the manufacturer. Shortly, 1 μl 10 DNase I reaction buffer and 1 μl DNase I (1 U/μl) were added per 10 μl of DEPC-treated water used for the re-suspension of the RNA pellet, and RNA samples were incubated for 15 min at room temperature. DNase was inactivated by addition of 1 μl of 25 mM EDTA and heating at 65 °C for 10 min, and was then removed through a second precipitation step. Following the addition of 10 μg Glycogen/mL TRIzol®, the tube was filled with RNase−/DNase-free water to 100 μL. Thereafter, 10 μL 3 M NaOAc (Sigma-Aldrich, Buchs, Switzerland) and 100 μL 100% isopropanol (Sigma-Aldrich, Buchs, Switzerland) were added at 1/10 volume. After incubation of samples at room temperature for 45 min, the washing steps with 75% ethanol (Sigma-Aldrich, Buchs, Switzerland) were twice performed as described above. Finally, the RNA pellet was suspended in 10 μL of RNase−/DNase-free water (Gibco, Life Technologies), incubated for 10 min on ice and right after for 10 min at 55 °C before being used for downstream analysis.

##### Total RNA quality control

Each sample was subjected to quality control regarding the quantity, purity and integrity of harvested RNA, using spectrometric evaluation (NanoDrop® 3300 Fluorospectrometer, ThermoFisher Scientific Inc., V 2.8 software, U.S.A.), and an electrophoretic assay (Agilent RNA 6000 Pico Kit and Agilent BioAnalyzer 2100 system, Agilent Technologies, Santa Clara CA, U.S.A.). RNA samples of both fertility groups showed comparable total RNA concentration, i.e. 1.6 ± 1.1 ng/μL and 1.6 ± 1.2 ng/μL for the LF and HF group, respectively. The BioAnalyzer electropherograms of the examined samples showed no 18S or 28S peaks that would be indicative of somatic cell contamination; a representative electropherogram is presented in Additional file 8, Figure C. The isolated total RNA samples were stored at − 80 °C until used for library preparation.

#### Small RNA library preparation

Using a total RNA input of 1 ng, small RNA libraries were prepared with the NEXTflex® Small RNA Sequencing Kit v3 for Illumina® Platforms (Bioo Scientific, Austin TX, U.S.A) according to the manufacturer’s instructions. A sufficient amount of ~ 150 bp product was detected after 22 cycles of polymerase chain reaction amplification, but also a considerable amount of ~ 130 bp adapter-only product was observed in the analysis of the small RNA libraries with an Agilent High Sensitivity DNA Kit (Agilent Technologies, Santa Clara CA, U.S.A.). To reduce the amount of adapter-only products, a polyacrylamide gel size selection was performed. A hand-cast 10% polyacrylamide gel was prepared with the following reagents: 0.8 mL of 50× Tris-acetate-EDTA (TAE), 29.2 mL of H_2_O, 0.25 g ammonium persulfate (APS) 25% dissolved in 1 mL H_2_O, 32 mL of N,N,N,N′-tetramethylenediamine (TEMED) which acts as a catalyst and 10 mL of 40% acrylamide. In a next step, all the reagents were combined in a beaker for the gel solution, except for the TEMED and APS that were degassed for 15 min. Just prior to pouring, TEMED and APS were added to the solution to polymerize in a prepared cast tray of two glass plates and two spacers. The solution was poured 1 cm bellow the teeth of the comb and it was left to cast for 1 h. After 1 h, when it is solid, the comb was removed and positioned to stand in the gel stand and 1× TAE buffer was poured. Gel wells were washed with a pipette. Then, 5 μL of 10 bp (2 μg/lane) of ready-to-load Low range DNA ladder (Gibco BRL, Life Technologies), 18 μL of sample and 2 μL of 6× loading dye (ThermoFisher Scientific Inc., Waltham, U.S.A.) were loaded. Samples were loaded slowly and allowed to settle evenly on the bottom of the well. After loading the samples, the gel was started at 100 V and left to run overnight. Then, the voltage was increased up to 300 V for 1 h until the dye bands reached the bottom of the gel. After 1 h, the gel was removed carefully from the glass plate and stained with SYBR Gold. Forty milliliters of 1× TAE and 4 μL SYBR Gold were prepared. The gel was stained on a shaker for 1 h. The area between 147 and 165 bp was cut out on a UV transilluminator (ChemiDoc™ MP Imaging System, Bio-Rad) using a clean scalpel. The gel slices were placed into a clean 1.5 mL tube and crushed thoroughly with a disposable pestle.

##### Purification from the gel slice

300 μL of elution buffer were added to the crushed gel slices. To obtain as much gel as possible, the pestle was also washed in the tube with the buffer. Washed gel slices were incubated around 3.5 h at room temperature on a shaker at 400 rpm. The eluate including crushed gel was transferred carefully to spin columns (Millipore Centrifugal Filter units). After centrifugation at 16,000 rcf for 2 min, spin filters were disposed. Then, 50 μL of NEXTflex Cleanup Beads and 350 μL of isopropanol were added and incubated at room temperature for 10 min, vortexed and spinned down for 10 s. The supernatant was separated in clean tubes and magnetized for 2 min or until the solution appeared clear. After discarding the supernatant, 950 μL of freshly prepared 80% ethanol was added and incubated for 30 s, then the supernatant was removed. This washing step was repeated twice and thereafter samples were dried for 3 min. Then, samples were removed from the magnetic stand and bead pellets were re-suspended in 13 μL of resuspension buffer. Finally, the samples were incubated for 2 min and magnetized for 3 min. Thereafter, 12 μL of supernatant, which was the sequencing library, were transferred to clean 1.5 mL tubes.

#### Sequencing and bioinformatics analysis

Small RNA libraries were sequenced at the Functional Genomics Center Zurich (Zurich, Switzerland) as one pool of 18 barcode-tagged samples on one lane of an Illumina HiSeq 2500 instrument (75 bp single-end reads). Analysis of the obtained fastq files was performed on a local Galaxy server installation [90] as previously described [91, 92]. Briefly, the tool ‘clip adapter sequences’ was used to remove adapter sequences. Non-clipped sequences were discarded. Removal of PCR duplicates was based on the four random nucleotides on each side of the cDNA inserts that were introduced during ligation of the 5′ and 3′ RNA adapters during library preparation. All identical cDNA sequences containing the same four nucleotides at the ends were removed by using the tool ‘collapse’. Afterwards, the four random bases were cut at each side of the sequences and unique sequences and their read counts were obtained by running the tool ‘collapse’ again. Then, a count table was generated by joining the lists of all samples in one table and read counts were scaled by total number of reads. The sequences with neglectable read counts, mainly derived from sequencing errors were removed using the cpm per sample filtering tool. Then, the cutoff was set to 42.84 cpm corresponding to an average of 20 reads per library for at least 5 out of 18 libraries. After that, the annotation of small RNA sequences was performed using Basic Local Alignment Search Tool (BLAST). The BLAST databases involved bovine and human sequences from miRBase (release 22.1), Rfam 14.1, transcript sequences from Ensembl and National Center for Biotechnology Information (NCBI), including non-coding RNAs, and predicted piRNA sequences.

The analysis of DE miRNAs was performed using the Bioconductor package EdgeR [93]. An adjusted *P* value (FDR) of 10% was used as a threshold to determine DE miRNAs. Subsequently, the MicroRNA ENrichment TURned NETwork (MIENTURNET; http://userver.bio.uniroma1.it/apps/mienturnet/) was used to identify target genes of two lists of miRNAs, the two DE miRNAs and the five miRNAs used for robust regression, with FDR < 5%,. The miRNA-target interactions were provided from the miRTarBase database via the MIENTURNET tool [94]. In addition, following the identification of target genes, the biological DataBase network bioDBnet (http://biodbnet.abcc.ncifcrf.gov) allowed us to match gene symbols with Entrez Gene IDs (bovine and putative homolog orthologs) [95]. Gene ontology enrichment analysis (biological process, molecular function, cellular localization) and pathway analysis were conducted using the Metascape web platform (http://metascape.org/gp/index.html#/main/step1) [96]. For each given gene list, pathway and process enrichment analysis was carried out with the following ontology sources: KEGG Functional Sets, GO Biological Processes, KEGG Pathway, GO Molecular Functions, GO Cellular Components, KEGG Structural Complexes, Reactome Gene Sets, Canonical Pathways, BioCarta Gene Sets, CORUM, TRRUST and Transcription Factor Targets. All genes in the genome have been used as the enrichment background. Terms with a P value < 0.01, a minimum count of 3, and an enrichment factor (i.e. the ratio between the observed counts and the counts expected by chance) > 1.5 were collected and grouped into clusters based on their membership similarities. More specifically, *P* values were calculated based on the accumulative hypergeometric distribution [97], and q-values were calculated using the Benjamini-Hochberg procedure to account for multiple testing [98]. Kappa scores [99] were used as the similarity metric when performing hierachical clustering on the enriched terms, and sub-trees with a similarity of > 0.3 were considered a cluster. The most statistically significant term within a cluster was chosen to represent the cluster. The functional network of the representative enriched GO terms was visualized using the Cytoscape software (v3.1.2) [100] with “force-directed” layout and edge bundled for clarity.

### Statistical analysis

Three out of the 18 bulls (two LF and one HF bulls) were not included in the statistical analysis because of their outlier miRNA expression levels. The statistical analysis was performed using the R Language for Statistical Computing version 3.6.1 [101].

#### Descriptive statistics

The set of continuous variables that was used as input for statistical analysis included: a) sperm quality traits (progressive motility, %DFI, PMAI sperm, C_pos_PI_neg_PNA_neg_F_neg_M_pos_ sperm), b) fertility measures (NRR_conv_, NRR_ss_, Δ_NRR_), and c) miRNA expression levels (cpm). The mean value, standard deviation, min and max values were reported as descriptive measures of sperm quality and fertility traits. The association between miRNA expression levels and sperm quality traits or fertility variables was tested using the Spearman’s rank correlation coefficient r_s_; the respective P values were corrected for multiple testing using the Holm-Bonferroni method. In this case, r_s_ values were computed at bull level, i.e. between the cpm of individual miRNAs and the mean value of sperm quality traits across ejaculates of the same bull. The Spearman’s correlation test was carried out on the overall dataset, including data of both experimental groups.

#### Principal component analysis

The expression levels of the 85 identified miRNAs were assumed to be linearly inter-correlated; thus, PCA was performed to summarize and visualize the variance of the miRNA expression dataset. The standardization of miRNA expression levels, extraction of principal components and graphical demonstration of the PCA output was performed using the toolset of the *FactoMineR* [102] and *factoextra* [103] statistical packages for R.

#### Robust regression

Forward model selection was employed to identify the subset of miRNAs whose expression made the most valuable contribution to explaining the variance of NRR_ss_. The selection process was performed twice using two different datasets: a dataset including all 85 miRNAs detected in the examined sperm samples, and a dataset including only the 72 miRNAs with the highest contribution to the first 5 PCs (Additional file 3, Table S7). In both cases, the same subset of miRNAs was recovered. Thus, a series of linear models including a maximum of five predictors were built using the *regsubsets* function in the *leaps* package for R [104]. The linear model with the best overall fit was selected based upon the values of the Bayesian Information Criterion. After determining the five most important miRNAs, the relationship between NRR_ss_ and the latter was assessed with robust regression. The robust regression approach for RNA-seq data has been suggested by Seo et al. (2016) as a way to overcome the issue of influential observations in experiments of small sample size [105]. Shortly, the *rlm* function of the *MASS* package for R was applied for fitting the regression line with an MM-estimator [106], with the expression levels of the selected miRNAs and NRR_ss_ functioning as predicting and response variables of the model M_ss_, respectively. The use of MM-estimation, a combination of S- and M-estimators, is robust against and allows the detection of outliers in the dataset. Because of the small sample size in our study, model parameters were determined using the Wald test. Similar to model M_ss_, a second robust regression line (M_conv_) was fit with the expression levels of the five selected miRNAs functioning as predictors of NRR_conv_. Values of the outcome and predicting variables in M_ss_ and M_conv_ were mean-centered and scaled by one SD, in order to compare the standardized b coefficients between the two models.

## Supplementary Information

**Additional file 1: Table S1.** Counts per million reads of 15 small RNA libraries.

**Additional file 2: Table S2.** Spearman’s rank correlation coefficients and adjusted *P* values.

**Additional file 3: Table S3.** Coordinates (i.e. correlation) of miRNA expression on the first five principal components. **Table S4.** Quality of representation of each miRNA (expressed as a squared cosine) for each of the first five principal components. **Table S5.** Contribution of 85 miRNAs to the first five principal components. **Table S6.** The amount of variation explained by each of the 14 principal components expressed as eigenvalue, percentage of total variance and cumulative percentage. **Table S7.** MiRNAs whose expression levels significantly correlated with at least one of the first principal components.

**Additional file 4: Table S8.** Potential target genes of differentially expressed miRNAs. **Table S9.** GO terms for the target genes of differentially expressed miRNAs. **Table S10.** Potential target genes of regression predictor miRNAs. **Table S11.** GO terms for the potential target genes of the regression predictor miRNAs.

**Additional file 5: Figure A and B.** Functional network of the most representative GO terms of the top 20 clusters (biological processes) identified as potential targets of the differentially expressed miRNAs, colored by cluster and P value, respectively.

**Additional file 6.** Minimum information on flow cytometric experiment. The Sperm Chromatin Structure Assay™.

**Additional file 7.** Minimum information on flow cytometric experiment. The five-color flow cytometric assay for sperm quality assesment.

**Additional file 8: Figure C.** Representative BioAnalyzer electropherogram of total RNA extracted from cryopreserved bovine sperm.

## Data Availability

The bulls included in this study as well as the biological material and data (small RNA sequencing data, artificial insemination fertility data) are a property of Swissgenetics (Swissgenetics, Meielenfeldweg 12, CH-3052 Zollikofen BE, Switzerland; https://swissgenetics.com/). The small RNA sequencing data that support the findings of this study were used under license for this current study only. Data are, however, available from the authors upon reasonable request and with permission of Swissgenetics; restrictions apply to the availability of these data.

## Abbreviations

%DFI: Percentage of sperm with high DNA fragmentation index
AI: Artificial insemination
AM: Acetoxymethyl
AO: Acridine orange
APS: Ammonium persulfate
BLAST: Basic Local Alignment Search Tool
BP: Band-pass
CASA: Computer-assisted sperm analysis
cpm: Count per million
C_pos_PI_neg_PNA_neg_F_neg_M_pos_: percentage of sperm with high esterase activity, intact plasma membrane, unstained acrosome, low intracellular Ca^2+^ levels and high mitochondrial membrane potential
DAVID: Database for annotation, visualization and integrated discovery
DE: Differentially expressed
DEPC: Diethyl pyrocarbonate
DFI: DNA fragmentation index
DiIC_1_(5): 1,1′,3,3,3′,3′-Hexamethylindodicarbocyanine iodide
DMSO: Dimethyl sulfoxide
DTT: Dithiothreitol
EDTA: Ethylenediaminetetraacetic acid
FDR: False discovery rate
GO: Gene ontology
HF: High fertility
IGF2R: Insulin-like growth factor 2 receptor
KEGG: Kyoto Encyclopedia of Genes and Genomes
LF: Low fertility
M_conv_: Robust regression line for the prediction of the non-return rate of conventional sperm
MIENTURNET: MicroRNA Enrichment Turned Network
miRNA: microRNA
M_ss_: Robust regression line for the prediction of the non-return rate of sex-sorted sperm
NCBI: National Center for Biotechnology Information
NRR: Non-return rate
NRR_conv_: Non-return rate for conventional sperm
NRR_ss_: Non-return rate for sex-sorted sperm
PBS: Phosphate buffered saline
PC: Principal component
PCA: Principal component analysis
PCR: Polymerase chain reaction
PE-PNA: Phycoerythrin-conjugated agglutinin of *Arachis hypogea*
PI: Propidium iodide
piRNA: Piwi-interacting RNA
PMAI: Percentage of plasma membrane- and acrosome-intact sperm
rcf: Relative centrifugal force
r_s_: Spearman’s rank correlation coefficient
SCSA: Sperm chromatin structure assay
SD: Standard deviation
SDS: Sodium dodecyl sulfate
SEM: Standard error of the mean
sncRNA: Small non-coding RNA
TAE: Tris-acetate-EDTA
TEMED: N,N,N,N′-tetramethylenediamine
TNE: Tris-NaCl EDTA
VIF: Variance inflation factor
Δ_NRR_: Relative change of the non-return rate

## Footnotes

1 Technical details of the used equipment and the protocol followed for the performance of the SCSA are provided according to the suggestion of Lee et al. (2009) [86] in Additional file 6.

2 Technical details of the used equipment and the protocol followed for the performance of the multicolor assay are provided according to the suggestion of Lee et al. (2009) [86] in Additional file 7.

## References

[1] Seidel GEJ (2014). Update on sexed semen technology in cattle. Animal..

[2] Ettema JF, Østergaard S (2015). Short communication : economics of sex-biased milk production. J Dairy Sci.

[3] Osada M, Iwabuchi H, Aoki T, Sasaki K, Ushijima H, Ozawa T (2019). Economic evaluation of artificial insemination of sex-sorted semen on a Brown Swiss dairy farm-a case study. Anim Sci J.

[4] Hutchison JL, Bickhart DM (2016). Sexed-semen usage for Holstein AI in the United States. J Anim Sci.

[5] Heuer C, Kendall D, Sun C, Deeb J, Moreno J, Vishwanath R (2017). Evaluation of conception rates of sex-sorted semen in commercial dairy farms over the last five years. In: ADSA annual meeting.

[6] Rath D, Barcikowski S, De Graaf S, Garrels W, Grossfeld R, Klein S (2013). Sex selection of sperm in farm animals: status report and developmental prospects. Reproduction..

[7] Asma-ul-Husna AM, Mehmood A, Sultana T (2017). Sperm sexing in Nili-Ravi buffalo through modified swim-up : Validation using SYBR® green real-time PCR. Anim Reprod Sci.

[8] Umehara T, Tsujita N, Id MS. Activation of toll-like receptor 7/8 encoded by the X chromosome alters sperm motility and provides a novel simple technology for sexing sperm. PLoS Biol. 2019. 10.1371/journal.pbio.3000398. 10.1371/journal.pbio.3000398 PMC6691984 31408454

[9] Vishwanath R, Moreno JF (2018). Review : Semen sexing – current state of the art with emphasis on bovine species.

[10] Seidel GE, Schenk JL (2008). Pregnancy rates in cattle with cryopreserved sexed sperm: effects of sperm numbers per inseminate and site of sperm deposition. Anim Reprod Sci.

[11] DeJarnette JM, Nebel RL, Marshall CE (2009). Evaluating the success of sex-sorted semen in US dairy herds from on farm records. Theriogenology..

[12] Schenk JL, Cran DG, Everett RW, Seidel GE (2009). Pregnancy rates in heifers and cows with cryopreserved sexed sperm: effects of sperm numbers per inseminate, sorting pressure and sperm storage before sorting. Theriogenology..

[13] Chebel RC, Guagnini FS, Santos JEP, Fetrow JP, Lima JR (2010). Sex-sorted semen for dairy heifers : effects on reproductive and lactational performances. J Dairy Sci.

[14] Norman HD, Hutchison JL, Miller RH (2010). Use of sexed semen and its effect on conception rate, calf sex, dystocia, and stillbirth of Holsteins in the United States. J Dairy Sci.

[15] Butler ST, Hutchinson IA, Cromie AR, Shalloo L (2014). Applications and cost benefits of sexed semen in pasture-based dairy production systems. Animal..

[16] Seidel GEJ. Economics of selecting for sex : the most important genetic trait. 2003;59:585–98. 10.1016/s0093-691x(02)01242-6 12499006

[17] Olynk NJ, Wolf CA (2007). Expected net present value of pure and mixed sexed semen artificial insemination strategies in dairy heifers. J Dairy Sci.

[18] Lenz RW, Gonzalez-Marin C, Gilligan TB, DeJarnette JM, Utt MD, Helser LA (2017). SexedULTRA™, a new method of processing sex-sorted bovine sperm improves conception rates. Reproduction, Fertility and Development.

[19] Thomas JM, Locke JWC, Vishwanath R, Hall JB, Ellersieck MR, Smith MF (2017). Effective use of SexedULTRA™ sex-sorted semen for timed artificial insemination of beef heifers. Theriogenology..

[20] Thomas JM, Locke JWC, Bonacker RC, Knickmeyer ER, Wilson DJ, Vishwanath R (2019). Evaluation of SexedULTRA 4M™ sex-sorted semen in timed artificial insemination programs for mature beef cows. Theriogenology..

[21] Holden SA, Fernandez-Fuertes B, Murphy C, Whelan H, Gorman AO, Brennan L (2017). Relationship between *in vitro* sperm functional assessments, seminal plasma composition, and field fertility after AI with either non-sorted or sex-sorted bull semen. Theriogenology..

[22] Bodmer M, Janett F, Hässig M, Den Daas N, Reichert P, Thun R (2005). Fertility in heifers and cows after low dose insemination with sex-sorted and non-sorted sperm under field conditions. Theriogenology..

[23] Dejarnette JM, Leach MA, Nebel RL, Marshall CE, McCleary CR, Moreno JF (2011). Effects of sex-sorting and sperm dosage on conception rates of Holstein heifers: is comparable fertility of sex-sorted and conventional semen plausible?. J Dairy Sci.

[24] Norman HD, Hutchison JL, VanRaden PM (2011). Evaluations for service-sire conception rate for heifer and cow inseminations with conventional and sexed semen. J Dairy Sci.

[25] DeJarnette JM, Nebel RL, Marshall CE, Moreno JF, McCleary CR, Lenz RW (2008). Effect of sex-sorted sperm dosage on conception rates in Holstein heifers and lactating cows. J Dairy Sci.

[26] Dejarnette JM, Mccleary CR, Leach MA, Moreno JF, Nebel RL, Marshall CE (2010). Effects of 2.1 and 3.5 × 10^6^ sex-sorted sperm dosages on conception rates of Holstein cows and heifers. J Dairy Sci.

[27] Xu J, Guo Z, Nedambale TL, Zhang J, Schenk J, Moreno JF (2006). Developmental potential of vitrified Holstein cattle embryos fertilized *in vitro* with sex-sorted sperm. J Dairy Sci.

[28] Blondin P, Beaulieu M, Fournier V, Morin N, Crawford L, Madan P (2009). Analysis of bovine sexed sperm for IVF from sorting to the embryo. Theriogenology..

[29] Inaba Y, Abe R, Geshi M, Matoba S, Nagai T, Somfai T (2016). Sex-sorting of spermatozoa affects developmental competence of *in vitro* fertilized oocytes in a bull-dependent manner. J Reprod Dev.

[30] Den Daas JHG, De Jong G, Lansbergen LMTE, Van Wagtendonk-De Leeuw AM (1998). The relationship between the number of spermatozoa inseminated and the reproductive efficiency of individual dairy bulls. J Dairy Sci.

[31] Dejarnette JM, Nebel RL, Marshall CE (2010). Understanding estimates of AI sire fertility. In: 23^rd^ technical conference on Artificial Insemination & Reproduction.

[32] Carvalho JO, Michalczechen-Lacerda VA, Sartori R, Rodrigues FC, Bravim O, Franco MM (2012). The methylation patterns of the *IGF2* and *IGF2R* genes in bovine spermatozoa are not affected by flow-cytometric sex sorting. Mol Reprod Dev.

[33] Seidel GE. Sexing mammalian sperm – Where do we go from here ? 2012;58:505–9. 10.1262/jrd.2012-077 23124700

[34] Dai D, Qazi IH, Ran M, Liang K, Zhang Y (2019). Exploration of miRNA and mRNA profiles in fresh and frozen-thawed boar sperm by transcriptome and small RNA sequencing. Int J Mol Sci.

[35] Zeng C, Peng W, Ding L, He L, Zhang Y, Fang D (2014). A preliminary study on epigenetic changes during boar spermatozoa cryopreservation. Cryobiology..

[36] Morton KM, Herrmann D, Sieg B, Struckmann C, Maxwell WMC, Rath D (2007). Altered mRNA expression patterns in bovine blastocysts after fertilisation *in vitro* using flow-cytometrically sex-sorted sperm. Mol Reprod Dev.

[37] Beilby KH, de Graaf SP, Evans G, Maxwell WMC, Wilkening S, Wrenzycki C (2011). Quantitative mRNA expression in ovine blastocysts produced from X- and Y-chromosome bearing sperm, both *in vitro* and *in vivo*. Theriogenology..

[38] Reza AMMT, Choi YJ, Han SG, Song H, Park C, Hong K (2019). Roles of microRNAs in mammalian reproduction: from the commitment of germ cells to peri-implantation embryos. Biol Rev.

[39] Robertson LR, Feugang JM, Rodriguez-Osorio N, Kaya A, Memili E (2008). 93 MicroRNA sequences of bul spermatozoa. Reprod Fertil Dev.

[40] Govindaraju A, Uzun A, Robertson L, Atli MO, Kaya A, Topper E (2012). Dynamics of microRNAs in bull spermatozoa. Reprod Biol Endocrinol.

[41] Du Y, Wang X, Wang B, Chen W, He R, Zhang L (2014). Deep sequencing analysis of microRNAs in bovine sperm. Mol Reprod Dev.

[42] Sellem E, Marthey S, Rau A, Jouneau L, Bonnet A, Perrier JP, et al. A comprehensive overview of bull sperm-borne small non-coding RNAs and their diversity across breeds. Epigenetics Chromatin. 2020. 10.1186/s13072-020-00340-0. 10.1186/s13072-020-00340-0 PMC7106649 32228651

[43] Malama E, Bauersachs S, Siuda M, Janett F, Bollwein H (2018). The relation between the functional status and miRNA profile of cryopreserved bovine semen. Proceedings of Bull Fertility Conference - Theory to Practice.

[44] Sellem E, Marthey S, Kiefer H, Le DC, Allais-Bonnet A, Jouneau L (2018). Bull sperm sncRNAs: A new source for potential fertility biomarkers?. Proceedings of Bull Fertility Conference - Theory to Practice.

[45] Menezes ESB, Badial PR, El Debaky H, Husna AU, Ugur MR, Kaya A (2020). Sperm miR-15a and miR-29b are associated with bull fertility. Andrologia..

[46] Zhang Y, Dai D, Chang Y, Li Y, Zhang M, Zhou G (2017). Cryopreservation of boar sperm induces differential microRNAs expression. Cryobiology..

[47] Li Y, Li RH, Ran MX, Zhang Y, Liang K, Ren YN (2018). High throughput small RNA and transcriptome sequencing reveal capacitation-related microRNAs and mRNA in boar sperm. BMC Genomics.

[48] Fraser L, Brym P, Pareek CS, Mogielnicka-Brzozowska M, Paukszto, Jastrzębski JP (2020). Transcriptome analysis of boar spermatozoa with different freezability using RNA-Seq. Theriogenology..

[49] Sosa E, Flores L, Yan W, McCarrey JR (2015). Escape of X-linked miRNA genes from meiotic sex chromosome inactivation. Dev..

[50] Turner JMA (2007). Meiotic sex chromosome inactivation. Development..

[51] Tscherner A, Gilchrist G, Smith N, Blondin P, Gillis D, LaMarre J (2014). MicroRNA-34 family expression in bovine gametes and preimplantation embryos. Reprod Biol Endocrinol.

[52] Capra E, Turri F, Lazzari B, Cremonesi P, Gliozzi TM, Fojadelli I, et al. Small RNA sequencing of cryopreserved semen from single bull revealed altered miRNAs and piRNAs expression between high- and low-motile sperm populations. BMC Genomics. 2017. 10.1186/s12864-016-3394-7. 10.1186/s12864-016-3394-7 PMC5209821 28052756

[53] Yuan S, Tang C, Zhang Y, Wu J, Bao J, Zheng H (2015). Mir-34B/C and mir-449a/B/C are required for spermatogenesis, but not for the first cleavage division in mice. Biol Open.

[54] Das PJ, McCarthy F, Vishnoi M, Paria N, Gresham C, Li G, et al. Stallion sperm transcriptome comprises functionally coherent coding and regulatory RNAs as revealed by microarray analysis and RNA-seq. PLoS One. 2013. 10.1371/journal.pone.0056535. 10.1371/journal.pone.0056535 PMC3569414 23409192

[55] Chen X, Che D, Zhang P, Li X, Yuan Q, Liu T (2017). Profiling of miRNAs in porcine germ cells during spermatogenesis. Reproduction..

[56] Nixon B, Stanger SJ, Mihalas BP, Reilly JN, Anderson AL, Holt JE, et al. The microRNA signature of mouse spermatozoa is substantially modified during epididymal maturation. 2015. 10.1095/biolreprod.115.132209. 10.1095/biolreprod.115.132209 26333995

[57] Krawetz SA, Kruger A, Lalancette C, Tagett R, Anton E, Draghici S (2011). A survey of small RNAs in human sperm. Hum Reprod.

[58] Abu-Halima M, Hammadeh M, Backes C, Fischer U, Leidinger P, Lubbad AM (2014). Panel of five microRNAs as potential biomarkers for the diagnosis and assessment of male infertility. Fertil Steril.

[59] Pantano L, Jodar M, Bak M, Ballesca JL, Tommerup N, Oliva R, et al. The small RNA content of human sperm reveals pseudogene-derived piRNAs complementary to protein-coding genes. 2015;21:1085–95. 10.1261/rna.046482.114 PMC4436662 25904136

[60] Stowe HM, Calcatera SM, Dimmick MA, Andrae JG, Duckett SK, Pratt SL. The bull sperm microRNAome and the effect of fescue toxicosis on sperm microRNA expression. PLoS One. 2014. 10.1371/journal.pone.0113163. 10.1371/journal.pone.0113163 PMC4251976 25462855

[61] Bouhallier F, Allioli N, Lavial F, Chalmel F, Perrard MH, Durand P (2010). Role of miR-34c microRNA in the late steps of spermatogenesis. RNA..

[62] Fang L, Sørensen P, Sahana G, Panitz F, Su G, Zhang S (2018). MicroRNA-guided prioritization of genome-wide association signals reveals the importance of microRNA-target gene networks for complex traits in cattle. Sci Rep.

[63] Long JE, Chen HX (2009). Identification and characteristics of cattle microRNAs by homology searching and small RNA cloning. Biochem Genet.

[64] Li R, Beaudoin F, Ammah AA, Bissonnette N, Benchaar C, Zhao X, et al. Deep sequencing shows microRNA involvement in bovine mammary gland adaptation to diets supplemented with linseed oil or safflower oil. BMC Genomics. 2015. 10.1186/s12864-015-1965-7. 10.1186/s12864-015-1965-7 PMC4628385 26519053

[65] Liu W-M, Pang RTK, Chiu PCN, Wong BPC, Lao K, Lee K-F (2012). Sperm-borne microRNA-34c is required for the first cleavage division in mouse. Proc Natl Acad Sci.

[66] Xiong C (2014). Identification of microRNAs predominately derived from testis and epididymis in human seminal plasma. Clin Biochem.

[67] Muñoz X, Mata A, Bassas L, Larriba S. Altered miRNA signature of developing germ-cells in infertile patients relates to the severity of spermatogenic failure and persists in spermatozoa. Sci Rep. 2015. 10.1038/srep17991. 10.1038/srep17991 PMC4673613 26648257

[68] Li X, Chen Y, Josson S, Mukhopadhyay NK, Kim J, Freeman MR (2013). MicroRNA-185 and 342 inhibit tumorigenicity and induce apoptosis through blockade of the SREBP metabolic pathway in prostate cancer cells.

[69] Cross NL. Minireview. Role of Cholesterol in Sperm Capacitation1 Nicholas. 1998;11:7–11. 10.1095/biolreprod59.1.7 9674986

[70] Travis AJ, Kopf GS (2002). The role of cholesterol efflux in regulating the fertilization potential of mammalian spermatozoa. J Clin Invest.

[71] Bucci D, Galeati G, Tamanini C, Vallorani C, Rodriguez-gil JE, Spinaci M (2012). Effect of sex sorting on CTC staining, actin cytoskeleton and tyrosine phosphorylation in bull and boar spermatozoa. Theriogenology..

[72] Carvalho JOO, Sartori R, Machado GMM, Mourão GBB, Dode MAN (2010). Quality assessment of bovine cryopreserved sperm after sexing by flow cytometry and their use in *in vitro* embryo production. Theriogenology.

[73] de Carvalho JO, Sartori R, Rodello L, Barreto G, Dimas S, Dode MAN (2018). Flow cytometry sex sorting affects bull sperm longevity and compromises their capacity to bind to oviductal cells. Livest Sci.

[74] Le Guillou S, Marthey S, Laloë D, Laubier J, Mobuchon L, Leroux C, et al. Characterisation and comparison of lactating mouse and bovine mammary gland miRNomes. PLoS One. 2014. 10.1371/journal.pone.0091938. 10.1371/journal.pone.0091938 PMC3962357 24658750

[75] Mobuchon L, Marthey S, Boussaha M, Le Guillou S, Leroux C, Le Provost F. Annotation of the goat genome using next generation sequencing of microRNA expressed by the lactating mammary gland: comparison of three approaches. BMC Genomics. 2015. 10.1186/s12864-015-1471-y. 10.1186/s12864-015-1471-y PMC4430871 25888052

[76] Tong M-H, Mitchell DA, McGowan SD, Evanoff R, Griswold MD (2012). Two miRNA clusters, mir-17-92 (mirc1) and mir-106b-25 (mirc3), are involved in the regulation of spermatogonial differentiation in mice1. Biol Reprod.

[77] Xie R, Lin X, Du T, Xu K, Shen H, Wei F, et al. Targeted disruption of miR-17-92 impairs mouse spermatogenesis by activating mTOR signaling pathway. Med (United States). 2016. 10.1097/MD.0000000000002713. 10.1097/MD.0000000000002713 PMC4998608 26886608

[78] Hurtado A, Real FM, Palomino R, Carmona FD, Burgos M, Jiménez R, et al. Sertoli cell-specific ablation of miR-17-92 cluster significantly alters whole testis transcriptome without apparent phenotypic effects. PLoS One. 2018. 10.1371/journal.pone.0197685. 10.1371/journal.pone.0197685 PMC5967698 29795630

[79] Kasimanickam V, Kastelic J. MicroRNA in sperm from Duroc, Landrace and Yorkshire boars. Sci Rep. 2016. 10.1038/srep32954. 10.1038/srep32954 PMC5011730 27597569

[80] Gao H, Wen H, Cao C, Dong D, Yang C, Xie S, et al. Overexpression of microrna-10a in germ cells causes male infertility by targeting rad51 in mouse and human. Front Physiol. 2019. 10.3389/fphys.2019.00765. 10.3389/fphys.2019.00765 PMC6591449 31275170

[81] Carvalho JO, Silva LP, Sartori R, Dode MAN. Nanoscale differences in the shape and size of X and Y chromosome-bearing bovine sperm heads assessed by atomic force microscopy. PLoS One. 2013. 10.1371/journal.pone.0059387. 10.1371/journal.pone.0059387 PMC3602057 23527178

[82] Suh TK, Schenk JL, Seidel GE (2005). High pressure flow cytometric sorting damages sperm. Theriogenology..

[83] Luna C, Li G, Qiu J, Epstein DL, Gonzalez P. MicroRNA-24 Regulates the processing of latent TGFβ1 during cyclic mechanical stress in human trabecular meshwork cells through direct targeting of FURIN. 2011;226:1407–14. 10.1002/jcp.22476 PMC3152464 20945401

[84] Hu B, Song JT, Qu HY, Bi CL, Huang XZ, Liu XX, et al. Mechanical stretch suppresses microRNA-145 expression by activating extracellular signal-regulated kinase 1/2 and upregulating angiotensin- converting enzyme to alter vascular smooth muscle cell phenotype. PLoS One. 2014. 10.1371/journal.pone.0096338. 10.1371/journal.pone.0096338 PMC4029552 24848371

[85] Yuan Y, Zhang L, Tong X, Zhang M, Zhao Y, Guo J (2017). Mechanical stress regulates bone metabolism through microRNAs. J Cell Physiol.

[86] Lee J, Spidlen J, Boyce K, Cai J, Crosbie N, Furlong J (2009). MIFlowCyt: the minimum information about a flow Cytometry experiment. Cytometry..

[87] Evenson D, Jost L. Sperm chromatin structure assay for fertility assessment. In: Current Protocols in Cytometry: John Wiley & Sons, Inc.; 2001. 10.1002/0471142956.cy0713s13. 10.1002/0471142956.cy0713s13 18770725

[88] Bucher K, Malama E, Siuda M, Janett F, Bollwein H (2019). Multicolor flow cytometric analysis of cryopreserved bovine sperm: a tool for the evaluation of bull fertility. J Dairy Sci.

[89] Rauber LP. Qualitative and quantitative analysis of porcine sperm transcripts and characterization of a normalized cDNA library. Ludwig-Maximilians-Universität München, Germany (Dissertation) https://edoc.ub.uni-muenchen.de/9368/1/Rauber_Lucio.pdf. Accessed 15 February 2020.

[90] Blankenberg D, Von Kuster G, Coraor N, Ananda G, Lazarus R, Mangan M, et al. Galaxy: A web-based genome analysis tool for experimentalists. Curr Protoc Mol Biol. 2010. 10.1002/0471142727.mb1910s89. 10.1002/0471142727.mb1910s89 PMC4264107 20069535

[91] Bick JT, Flöter VL, Robinson MD, Bauersachs S, Ulbrich SE. Small RNA-seq analysis of single porcine blastocysts revealed that maternal estradiol-17beta exposure does not affect miRNA isoform (isomiR) expression. BMC Genomics. 2018. 10.1186/s12864-018-4954-9. 10.1186/s12864-018-4954-9 PMC6090871 30081835

[92] Almiñana C, Tsikis G, Labas V, Uzbekov R, da Silveira JC, Bauersachs S, et al. Deciphering the oviductal extracellular vesicles content across the estrous cycle: implications for the gametes-oviduct interactions and the environment of the potential embryo. BMC Genomics. 2018. 10.1186/s12864-018-4982-5. 10.1186/s12864-018-4982-5 PMC6103977 30134841

[93] Robinson MD, Mccarthy DJ, Smyth GK. edgeR : a Bioconductor package for differential expression analysis of digital gene expression data. 2010;26:139–40. 10.1093/bioinformatics/btp616 PMC2796818 19910308

[94] Licursi V, Conte F, Fiscon G, Paci P (2019). MIENTURNET: an interactive web tool for microRNA-target enrichment and network-based analysis. BMC Bioinformatics.

[95] Mudunuri U, Che A, Yi M, Stephens RM (2009). bioDBnet: the biological database network. Bioinformatics..

[96] Zhou Y, Zhou B, Pache L, Chang M, Khodabakhshi AH, Tanaseichuk O, Benner C, Chanda SK (2019). Metascape provides a biologist-oriented resource for the analysis of systems-level datasets. Nat Commun.

[97] Zar, J.H. Biostatistical Analysis 1999 4^th^ edn., NJ Prentice Hall, pp. 523. ISBN-13 9780130815422.

[98] Hochberg Y, Benjamini Y (1990). More powerful procedures for multiple significance testing. Stat Med.

[99] Cohen J (1960). A coefficient of agreement for nominal scales. Educ Psychol Meas.

[100] Shannon P, Markiel A, Ozier O, Baliga NS, Wang JT, Ramage D, Amin N, Schwikowski B, Ideker T (2003). Cytoscape: a software environment for integrated models of biomolecular interaction networks. Genome Res.

[101] The R Development Core Team. A language and environment for statistical computing. R Foundation for Statistical Computing. 2019. http://www.r-project.org/. Accessed 15 February 2020.

[102] Le S, Josse J, Husson F (2008). FactoMineR: An R Package for multivariate analysis. J Stat Software.

[103] Kassambara A, Mundt F. factoextra: Extract and visualize the results of multivariate data analyses. 2020; R package version 1.0.7. https://CRAN.R-project.org/package=factoextra. Accessed 15 Sep 2020.

[104] Lumley T, Miller (based on Fortran code by Alan Miller). leaps: Regression Subset Selection. R package version 3.1. 2020. https://cran.r-project.org/web/packages/leaps/index.html. Accessed 6 Feb 2020.

[105] Seo M, Kim K, Yoon J, Jeong JY, Lee H, Cho S, et al. RNA-seq analysis for detecting quantitative trait-associated genes. Nat Publ Gr. 2016. 10.1038/srep24375. 10.1038/srep24375 PMC4829873 27071914

[106] Venables WN, Ripley BD (2002). Modern applied statistics with S.

