# Supplementary material for: The micro-RNA content of unsorted cryopreserved bovine sperm and its relation to the fertility of sperm after sex-sorting
Source: BMC Genomics. 2021 Jan 7;22:30. doi: 10.1186/s12864-020-07280-9 (PMC7792310; doi:10.1186/s12864-020-07280-9)
Supplement: Supplementary file 5 — Additional file 5: Figure A and B. Functional network of the most representative GO terms of the top 20 clusters (biological processes) identified as potential targets of the differentially expressed miRNAs, colored by cluster and P value, respectively. [file 12864_2020_7280_MOESM5_ESM.docx]

**Figure A**. Functional network of the most representative GO terms of the top 20 clusters (biological processes) identified as potential targets of the differentially expressed miRNAs, miR-9-5p and miR-10a-5p.


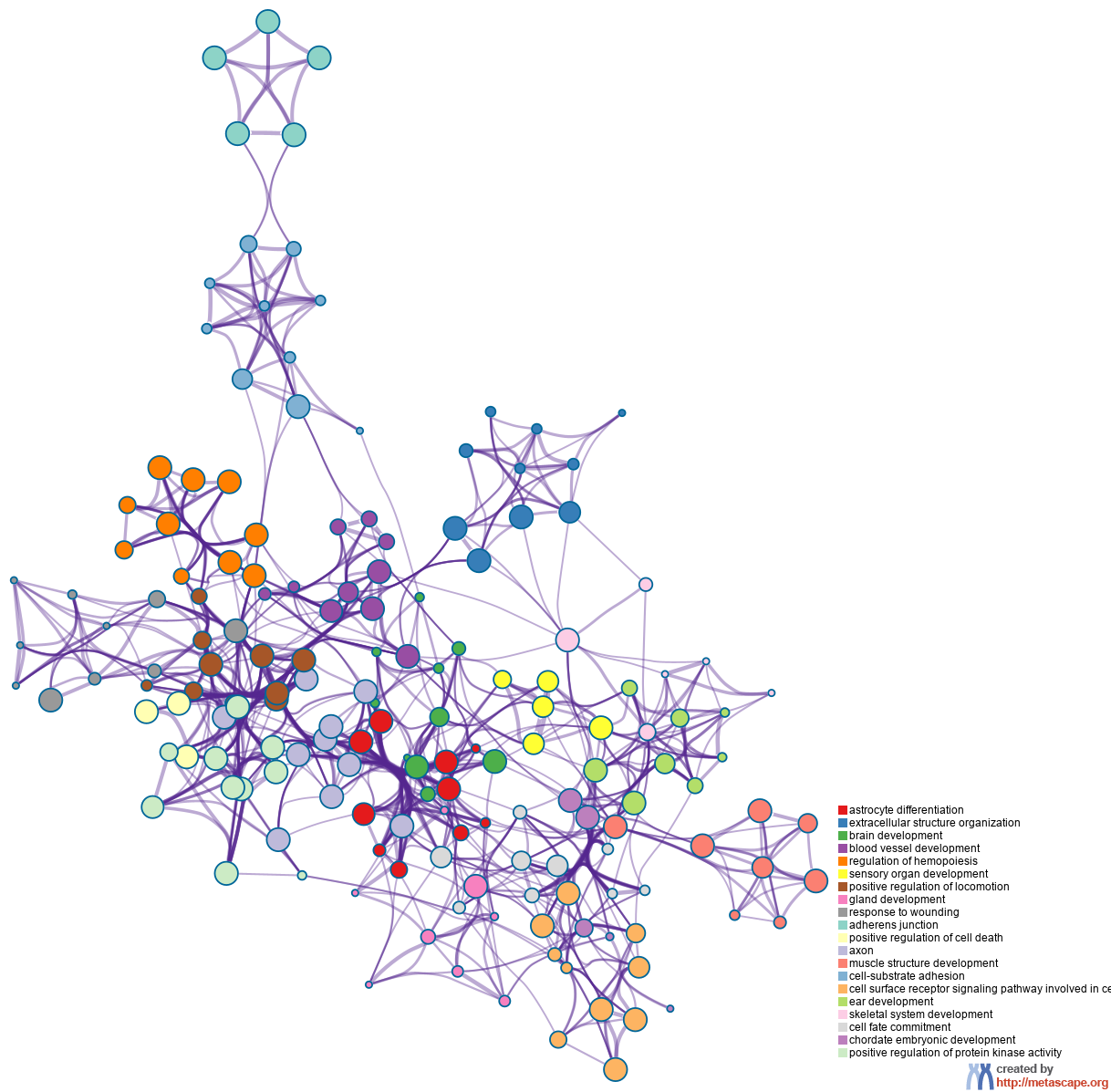


Each term was represented by a circle node, with its size being proportional to the number of input genes falling into this term, and its color being representive of its cluster identity (i.e., nodes of the same color belong to the same cluster). Terms with a similarity score >0.3 were linked by an edge (the thickness of the edge represented the similarity score).

**Figure B**. Functional network of the most representative GO terms (described in Figure A above) of the top 20 clusters (biological processes) identified as potential targets of the differentially expressed miRNAs, miR-9-5p and miR-10a-5p.


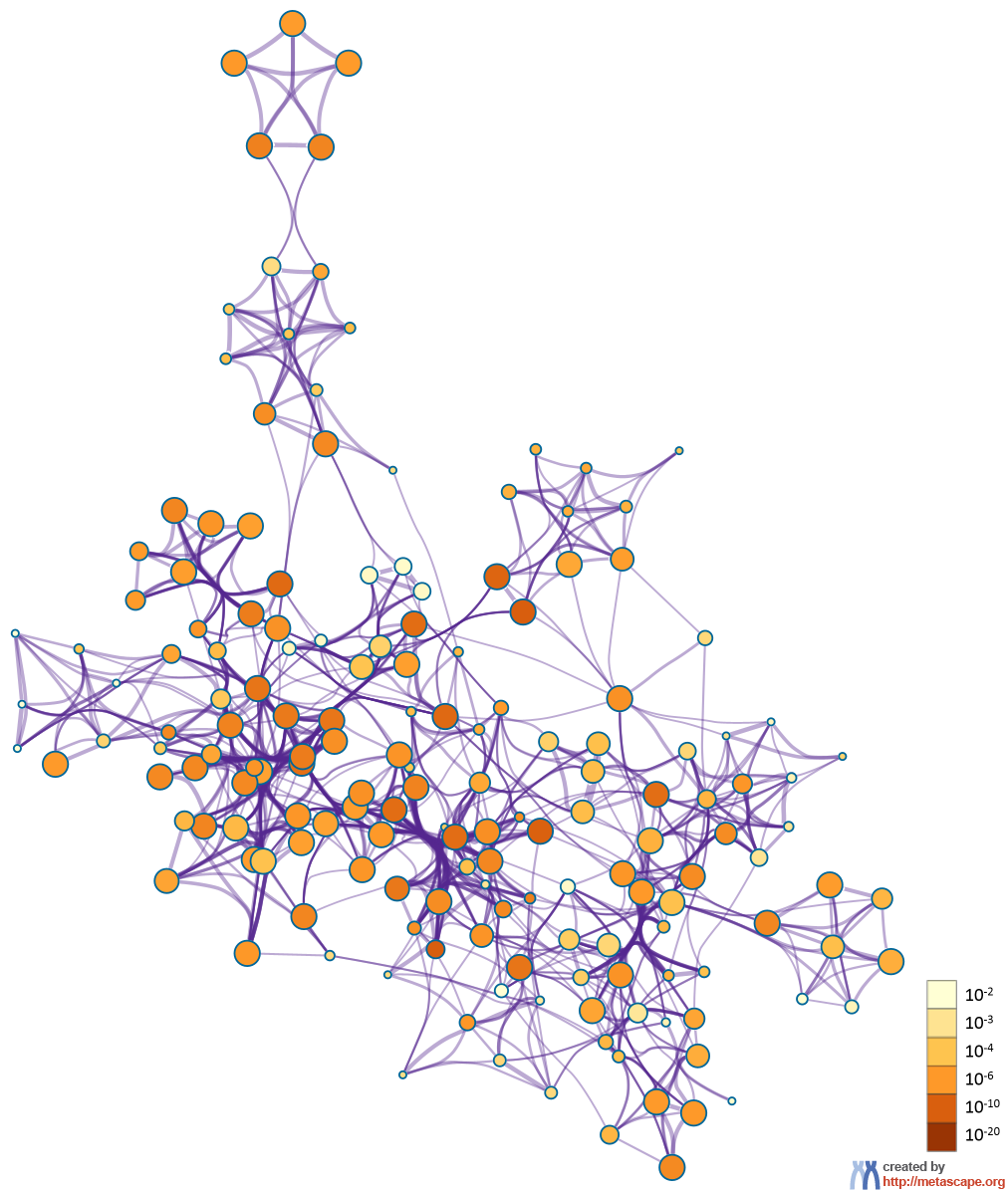


Each term was represented by a circle node, with its size being proportional to the number of input genes falling into this term, and its color being representive of its P value (i.e., nodes of darker color show higher significance). Terms with a similarity score >0.3 were linked by an edge (the thickness of the edge represented the similarity score).
