# Supplementary material for: The micro-RNA content of unsorted cryopreserved bovine sperm and its relation to the fertility of sperm after sex-sorting
Source: BMC Genomics. 2021 Jan 7;22:30. doi: 10.1186/s12864-020-07280-9 (PMC7792310; doi:10.1186/s12864-020-07280-9)
Supplement: Supplementary file 6 — Additional file 6. Minimum information on flow cytometric experiment. The Sperm Chromatin Structure Assay™. [file 12864_2020_7280_MOESM6_ESM.docx]

**Minimum Information about Flow Cytometric Experiment**

**(SPERM CHROMATIN STRUCTURE ASSAY)**

**1. Experiment Overview**

**1.1. Purpose**

The present study aimed to evaluate the miRNA profile of unsorted cryopreserved bovine semen as predictor of the fertilizing ability of sperm after sex-sorting. Apart from a flow cytometric panel consisting of five colors, the integrity of nuclear sperm chromatin structure was also tested using the SPERM CHROMATIN STRUCTURE ASSAY (SCSA; Evenson, D., and L. Jost. 2001. Sperm Chromatin Structure Assay for Fertility Assessment. In Current Protocols in Cytometry, S13: 1-27, John Wiley & Sons, Inc., doi 10.1002/0471142956.cy0713s13).

**1.2. Keywords**

bull; sex-sorted sperm; fertility; microRNA

**1.3. Experiment Variables**

The SCSA parameters, i.e. mean DNA fragmentation index (DFI), SD of DFI, and the percentage of sperm with high DFI (%DFI), were determined in bulls with low and high fertility after sex-sorting (high vs low fertility group as conditional variable).

**1.4. Organization**

1.4.1. Name: Clinic of Reproductive Medicine, Vetsuisse Faculty, University of Zurich

1.4.2. Address: Winterthurerstrasse 260, CH-8057 Zurich, Switzerland

**1.5. Primary Contact**

1.5.1. Name: Eleni Malama

1.5.2. Email: [emalama@vetclinics.uzh.ch](mailto:emalama@vetclinics.uzh.ch)

**1.6. Date**

Sperm samples were collected and cryopreserved in the year 2015-2016. Flow cytometric analysis was performed in September 2018.

**1.7. Conclusions**

The analysis of the data revealed a wide array of miRNA in unsorted bovine sperm. Five miRNA (miR-34c, miR-342, miR-7859, miR-106b-5p, miR-92a) were highlighted as potential predictors of the reproductive performance of the bull after artificial insemination in the field with sex-sorted sperm. The relation of sperm miRNA and flow cytometrically assessed sperm quality traits was found weak

**1.8. Quality Control Measures**

- A reference sample of cryopreserved bovine sperm (obtained from a proven sperm donor with known sperm quality characteristics) was stained and analyzed in parallel to the experimental samples
- Double aliquots of experimental samples

**1.9. Other Relevant Experiment Information**

The performance of the SCSA and the analysis of the relevant flow cytometric data followed the protocol guidelines published by Evenson and Jost (2001; Evenson, D., and L. Jost. 2001. Sperm Chromatin Structure Assay for Fertility Assessment. In Current Protocols in Cytometry, S13: 1-27, John Wiley & Sons, Inc., doi 10.1002/0471142956.cy0713s13)

**2. Flow Sample / Specimen Details**

**2.1. Sample / Specimen Material Description**

2.1.1. Biological Samples

2.1.1.1. Biological Samples Description: Bovine ejaculates collected in artificial vagina; ejaculates were cryopreserved in liquid nitrogen (-196 °C) after dilution with commercial sperm extender and packaging in 0.25-ml plastic straws

2.1.1.2. Biological sample source description: *Bos taurus taurus*

2.1.1.3. Biological Sample Source Organism Description: Bovine ejaculates collected in artificial vagina; ejaculates were cryopreserved in liquid nitrogen (-196 °C) after dilution with commercial sperm extender and packaging in 0.25-ml plastic straws

- Taxonomy: *Bos taurus taurus*
- Age: >1.5 years old
- Gender: male
- Treatment: N/A
- Other Relevant Biological Sample Source Organism Information:

All animals were kept in a single artificial insemination center, thus, handled and fed in an identical manner. Based on their field fertility records obtained after >500 first services with unsorted and sex-sorted frozen-thawed semen per year, bulls were grouped as high or low fertility sires after sex-sorting.

2.1.2. Environmental Samples

N/A

2.1.3. Other Samples

N/A

**2.2. Sample Characteristics**

Expected/analyzed type of cells/particles: spermatozoa, debris

**2.3. Sample Treatment Description**

- - - - Cryopreserved sperm samples were thawed in waterbath (38 °C, 30 sec)
- Three straws per ejaculate were pooled in single laboratory tube
- Sperm samples were split in two aliquots. The first aliquot was diluted to a concentration of 1 to 2 $\times$ 10^6^ sperm/mL with pre-warmed (38 °) TNE buffer and tested with the SCSA at 0h post-thaw; the second aliquot was incubated (38 °C, 5% CO_2_) for 3 hours, and subsequently diluted and tested as the first aliquot.
- For aliquots tested at 0h and 3h, 400 μL of acid detergent solution were added to 200 μL of diluted semen and thoroughly mixed for 30 sec
- 1.2 mL of acridine orange (AO) staining solution were added (6.0 μg AO/mL AO staining buffer)
- Samples were flow cytometrically analyzed after exactly 3 min

**2.4. Fluorescence Reagent Description**

Each sample has been stained according to the following table:

| *Optical detector* | FL1 (525/20 BP) | FL3 (620/15 BP) |
| --- | --- | --- |
| *Reporter* | Acridine orange (AO) | Acridine orange (AO) |
| *Concentration* | 6.0 μg AO/mL AO staining buffer | 6.0 μg AO/mL AO staining buffer |
| *Manufacturer* | Polysciences Inc. | Polysciences Inc. |
| *Cat#* | 04539 | 04539 |
| *Sample* | Sperm | Sperm |
| *Analyte* | dsDNA | ssDNA |
| *Characteristic* | Integrity of nuclear chromatin structure | Integrity of nuclear chromatin structure |

AO staining buffer, 0.2 M Na_2_HPO_4_, 1 mM EDTA, 0.15 M NaCl, 0.1 M citric acid, pH 6.0

**3. Instrument Details**

**3.1. Instrument Manufacturer**

Beckman Coulter, Inc.

<https://www.beckmancoulter.com/>

**3.2. Instrument Model**

Beckman Coulter EPICS XL flow cytometer

<https://www.gmi-inc.com/product/beckman-coulter-epics-xl-flow-cytometer/>

Technical specification at <https://www.beckmancoulter.com/wsrportal/techdocs?docname=4237298CA>

**3.3. Instrument Configuration and Settings**

3.3.1. Flow cell and fluidics

The instrument has not been altered; hydrodynamic focusing

3.3.2. Light Sources

The instrument has not been altered; Air-cooled, software controlled, 15 mW, argon ion laser operating at 488 nm

3.3.3. Excitation Optics Configuration

The instrument has not been altered

3.3.4. Optical Filters

The instrument has not been altered; all filters were original and came with the instrument.

**4. Data Analysis Details**

**4.1. List-mode Data Files**

LMD files can be obtained by contacting Dr. Eleni Malama after this work has been published.

**4.2. Compensation Description**

N/A

**4.3. Data Transformation Details**

4.3.1. Purpose of Data Transformation

Graphical illustration and gating

4.3.2. Data Transformation Description

The following visualization settings have been used for gating:

- FSC and SSC: linear scale
- FL1, FL3: linear scale

4.3.3. Other Relevant Data Transformation Details

Data analysis was performed using the FCS EXPRESS 4 Flow Cytometry Research Edition (4.07.0005 version) software (De Novo Software, Glendale, U.S.A.)

**4.4. Gating (Data Filtering) Details**

The same gating strategy has been used for all data files.

4.4.1. Gate Description

The following gates were applied:

- Forward scatter (FSC) vs. side scatter (SSC) gate to define sperm cells
- FL3 vs. FL1 gate to exclude debris and define the sperm gate A (Figure S13)
- The alpha t (α_t_) parameter was computed according to the formula:

*α_t_ =* $\frac{red fluorescence}{total fluorescence} \times1,000$

where *total fluorescence = red + green fluorescence*

- All sperm fluorescence signals were gated in an α_t_ histogram (Figure S14, panel A) and an α_t_ vs. total fluorescence cytogram (Figure S14, panel B); from the former, the mean α_t_ value and its SD (i.e. the mean DFI and the SD of DFI of the sperm sample, respectively) were calculated
- The α_t_ histogram (Figure S14, panel A) was also used for the calculation of %DFI. A region (M1) that started at the right-hand side of the main peak of α_t_ distribution and went to the upper channel was defined. The left-hand boundary of the above mentioned region was set based on a previously examined reference sperm sample of known α_t_ distribution characteristics. Events with an α_t_ value within the M1 region were considered as cells out of the main population (COMPα_t_); the percentage of COMPα_t_ in the total population of gated sperm equals to %DFI

4.4.2. Gate Boundaries

Figure S13: *FL3 vs. FL1 gate to exclude debris and define the sperm gate A; red-colored events indicate the cells out of main population*

Figure S14: *Sperm fluorescence signals gated in an α_t_ histogram (panel A) and an α_t_ vs. total fluorescence cytogram (panel B) for the determination of mean DFI, SD of DFI, and %DFI; ; red-colored events in the cytogram of panel B indicate the cells out of main population*

**B**

**A**
