# Supplementary material for: The micro-RNA content of unsorted cryopreserved bovine sperm and its relation to the fertility of sperm after sex-sorting
Source: BMC Genomics. 2021 Jan 7;22:30. doi: 10.1186/s12864-020-07280-9 (PMC7792310; doi:10.1186/s12864-020-07280-9)

**Minimum Information about Flow Cytometric Experiment**

**(Five-color assay for sperm quality assessment)**

**1. Experiment Overview**

**1.1. Purpose**

The present study aimed to evaluate the miRNA profile of unsorted cryopreserved bovine semen as predictor of the fertilizing ability of sperm after sex-sorting.

**1.2. Keywords**

bull; sex-sorted sperm; fertility; microRNA

**1.3. Experiment Variables**

The percentage of sperm (within the total sperm population) with a) intact plasma membrane and unstained acrosome, and b) high esterase activity, intact plasma membrane, unstained acrosome, low intracellular Ca^2+^ levels and high mitochondrial membrane potential was determined in bulls with low and high fertility after artificial insemination with sex-sorted sperm (high vs low fertility groups as conditional variable).

**1.4. Organization**

1.4.1. Name: Clinic of Reproductive Medicine, Vetsuisse Faculty, University of Zurich

1.4.2. Address: Winterthurerstrasse 260, CH-8057 Zurich, Switzerland

**1.5. Primary Contact**

1.5.1. Name: Eleni Malama

1.5.2. Email: [emalama@vetclinics.uzh.ch](mailto:emalama@vetclinics.uzh.ch)

**1.6. Date**

Sperm samples were collected and cryopreserved in the year 2015-2016. Flow cytometric analysis was performed in September 2018.

**1.7. Conclusions**

The analysis of the data revealed a wide array of miRNA in unsorted bovine sperm. Five miRNA (miR-34c, miR-342, miR-7859, miR-106b-5p, miR-92a) were highlighted as potential predictors of the reproductive performance of the bull after artificial insemination in the field with sex-sorted sperm. The relation of sperm miRNA and flow cytometrically assessed sperm quality traits was found weak.

**1.8. Quality Control Measures**

- Daily verification of flow cytometer’s optical alignment and fluidics system using CytoFLEX Daily QC Fluorospheres (3 μM; <https://www.beckman.ch/reagents/coulter-flow-cytometry/qc-and-support-reagents/b53230>)
- A reference sample of cryopreserved bovine sperm (obtained from a proven sperm donor with known sperm quality characteristics) was stained and analyzed in parallel to the experimental samples
- Double aliquots of experimental samples
- Comparison of flow cytometric data with image obtained through fluorescence microscopy

**1.9. Other Relevant Experiment Information**

N/A

**2. Flow Sample / Specimen Details**

**2.1. Sample / Specimen Material Description**

2.1.1. Biological Samples

2.1.1.1. Biological Samples Description: Bovine ejaculates collected in artificial vagina; ejaculates were cryopreserved in liquid nitrogen (-196 °C) after dilution with commercial sperm extender and packaging in 0.25-ml plastic straws

2.1.1.2. Biological sample source description: *Bos taurus taurus*

2.1.1.3. Biological Sample Source Organism Description: Bovine ejaculate collected in artificial vagina; ejaculates were cryopreserved in liquid nitrogen (-196 °C) after dilution with commercial sperm extender and packaging in 0.25-ml plastic straws

- Taxonomy: *Bos taurus taurus*
- Age: >1.5 years old
- Gender: male
- Treatment: N/A
- Other Relevant Biological Sample Source Organism Information:

All animals were kept in a single artificial insemination center, thus, handled and fed in an identical manner. Based on their field fertility records obtained after >500 first services with frozen-thawed semen per year, bulls were grouped as high or low fertility sires after sex-sorting.

2.1.2. Environmental Samples

N/A

2.1.3. Other Samples

N/A

**2.2. Sample Characteristics**

Expected/analyzed type of cells/particles: spermatozoa, debris

**2.3. Sample Treatment Description**

- - - - Cryopreserved sperm samples were thawed in waterbath (38 °C, 30 sec)
- Three straws per ejaculate were pooled
- Sperm were diluted to a concentration of 1.2 $\times$ 10^6^ sperm/mL with pre-warmed (38 °) Tyrode’s solution at a final volume of 244.75 μL in a 250-μL reaction well of a 96-well plate
- Staining solution (5.25 mL) was added to diluted sperm in form of a master-mix of the five fluorescent probes (please see table below)
- Samples were flow cytometrically analyzed at after 0 and 3 hours of incubation (38 °C, 5% CO_2_)

**2.4. Fluorescence Reagent Description**

Each sample has been stained according to the following table:

| *Optical detector* | V450 (450/45 BP) | FITC (525/40 BP) | PE (585/42 BP) | PC5.5 (690/50 BP) | APC (660/20 BP) |
| --- | --- | --- | --- | --- | --- |
| *Reporter* | Calcein violet | Fluo-4 AM | PE-PNA | Propidium iodide | DiIC_1_(5) |
| *Concentration* | 1.21 μM | 2 μM | 1 mg/ml | 2.99 mM | 0.015 μM |
| *Manufacturer* | ThermoFischer Scientific | ThermoFischer Scientific | GeneTex | Sigma-Aldrich | ThermoFischer Scientific |
| *Cat#* | C34858 | F14201 | GTX01509 | 25535-16-4 | M34151 |
| *Sample* | Sperm | Sperm | Sperm | Sperm | Sperm |
| *Analyte* | Ubiquitous intracellular esterase | Intracellular Ca^2+^ | Outer acrosomal membrane | Nuclear DNA | Mitochondrial membrane |
| *Characteristic* | Intracellular esterase activity (viability) | Intracellular Ca^2+^ levels | Acrosomal status | Plasma membrane integrity (viability) | Mitochondrial membrane potential |
| *Targeted cells* | Calcein-positive (C_pos_) | Fluo-4 AM-negative (F_neg_) | PE-PNA-negative (PNA_neg)_ | Propidium iodide-negative (PI_neg_) | DiIC_1_(5)-positive (M_pos_) |

PE-PNA, phycoerythrin-conjugated agglutinin of *Arachis hypogaea*; DiIC_1_(5), **1,1’,3,3,3’,3’-Hexamethylindodicarbocyanine iodide**

**3. Instrument Details**

**3.1. Instrument Manufacturer**

Beckman Coulter, Inc.

<https://www.beckmancoulter.com/>

**3.2. Instrument Model**

CytoFLEX Flow Cytometer V5-B5-R3

<https://www.beckman.com/flow-cytometry/instruments/cytoflex/b53000>

Technical specification at <http://www.pedsresearch.org/uploads/blog/doc/Cytoflex-Manual.pdf>

**3.3. Instrument Configuration and Settings**

3.3.1. Flow cell and fluidics

The instrument has not been altered; alignment-free integrated optics quartz flow cell (420 μm x 180 μm ID) design with >1.3 numerical aperture

3.3.2. Light Sources

The instrument has not been altered; three-laser configuration

- violet laser, wavelength:405-nm laser device, 80 mW
  blue laser, wavelength: 488-nm, 50 mW
- red laser, wavelength:638-nm, 50 mW

3.3.3. Excitation Optics Configuration

The instrument has not been altered

3.3.4. Optical Filters

The instrument has not been altered; all filters are original and came with the instrument. The instrument was equipped with a set of wavelength division multiplexers (WDM). Each WDM is a unique detector array that corresponds to a different laser. Each WDM contains optical filters and detectors for detecting channel fluorescence or scatter from a particular laser.

**4. Data Analysis Details**

**4.1. List-mode Data Files**

FCS files can be obtained by contacting Dr. Eleni Malama after this work has been published.

**4.2. Compensation Description**

To address the problematic of spectral overlapping a compensation matrix was computed on each experimental day. For this purpose, a positive and a negative population for every single-color sample was used, i.e. frozen-thawed bovine sperm diluted with TRILADYL extender (Minitüb GmbH, Tiefenbach, Germany). In parallel, the stainability and autofluorescence of the examined extender was taken into account using the appropriate non-sperm extender controls. The instrument’s gain configuration for each color was adjusted in parallel. Compensation was performed post acquisition according to the following compensation matrix.

| Autofluorescence | Channel | -FITC% | -PE% | -PC5.5% | -APC% | -V450% |
| --- | --- | --- | --- | --- | --- | --- |
| 63.85 | FITC |  | 0.50 | 0.15 | 0.25 | 0.00 |
| 39.29 | PE | 84.05 |  | 17.25 | 0.00 | 0.00 |
| 6.23 | PC5.5 | 13.15 | 0.00 |  | 2.75 | 0.04 |
| 4.35 | APC | 0.00 | 0.00 | 0.00 |  | 3.87 |
| 5.85 | V450 | 0.00 | 0.00 | 0.00 | 0.00 |  |

**4.3. Data Transformation Details**

4.3.1. Purpose of Data Transformation

Graphical illustration and gating

4.3.2. Data Transformation Description

The default visualization settings of CytExpert Software for CytoFLEX version 2.1 have been used for gating:

- FSC and SSC: linear scale
- All fluorescence parameters: logarithmic scale

**4.4. Gating (Data Filtering) Details**

The same gating strategy has been used for all data files.

4.4.1. Gate Description

The following gates were applied for the identification of single sperm cells and sperm sub-populations with specific cellular characteristics:

- Forward scatter area (FSC-A) vs. side scatter area (SSC-A) gate to define sperm cells (Figure S1)
- FSC-A vs forward scatter height (FSC-H) gate (Figure S2, panel A) and SSC-A vs. SSC-H gate (Figure S2, panel B) to exclude doublets
- PE vs. PC5.5 gate to define PInegPNAneg (PMAI) sperm (Figure S3)
- V450 vs. APC gate to define CposMpos sperm (Figure S4); FITC vs PC5.5 gate applied to C_pos_M_pos_ sperm to define C_pos_PI_neg_F_neg_M_pos_ sperm (Figure S5); PE gate (histogram) applied to C_pos_PI_neg_F_neg_M_pos_ sperm to define C_pos_PI_neg_PNA_neg_F_neg_M_pos_ sperm

The positive as well as the negative sperm sub-populations were also checked through fluorescence histogram for single colors.

4.4.2. Gate Boundaries

Figure S1: *FSC-A vs. SSC-A gate to define sperm*

**
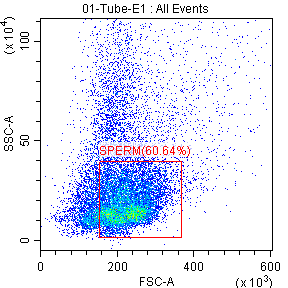
**

Figure S2: *FSC-A vs. FSC-H gate (panel A) and SSC-A vs. SSC-H gate (panel B) to discriminate doublets*

**
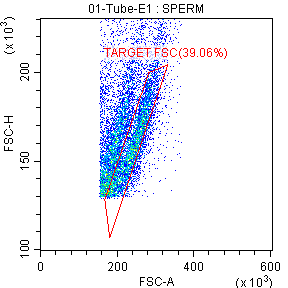
** **
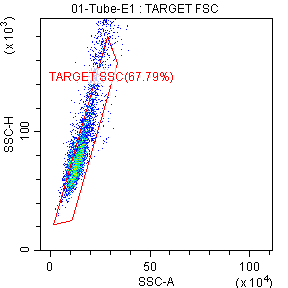
**

**B**

**A**

Figure S3: Figure S4:

*PE vs. PC5.5 gate to define PI_neg_PNA_neg_ sperm V450 vs. APC gate to define C_pos_M_pos_ sperm*


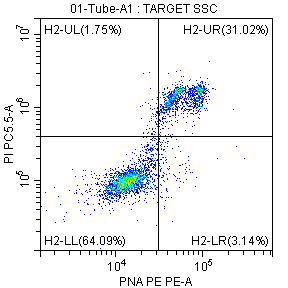

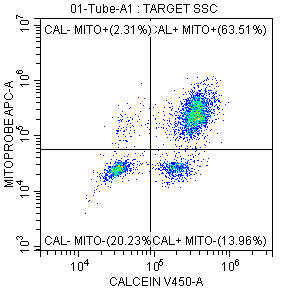


Figure S5:

*FITC vs. PC5.5 gate to define PI_neg_F_neg_ sperm*


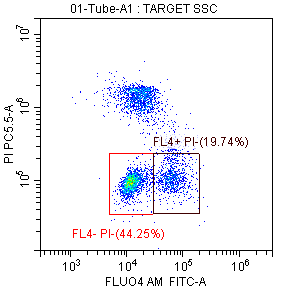

Supplement: Supplementary file 7 — Additional file 7. Minimum information on flow cytometric experiment. The five-color flow cytometric assay for sperm quality assesment. [file 12864_2020_7280_MOESM7_ESM.docx]
