# Supplementary material for: The micro-RNA content of unsorted cryopreserved bovine sperm and its relation to the fertility of sperm after sex-sorting
Source: BMC Genomics. 2021 Jan 7;22:30. doi: 10.1186/s12864-020-07280-9 (PMC7792310; doi:10.1186/s12864-020-07280-9)
Supplement: Supplementary file 8 — Additional file 8: Figure C. Representative BioAnalyzer electropherogram of total RNA extracted from cryopreserved bovine sperm. [file 12864_2020_7280_MOESM8_ESM.pdf]

Figure C. Representative BioAnalyzer electropherogram of total RNA extracted from cryopreserved bovine sperm. Electropherograms of the RNA ladder used for the assay (top panel) as well as the sperm total RNA sample (bottom panel) are presented.

2100 expert_Eukaryote Total RNA Pico_DEDAE00458_2018-05-07_15-46-02.xad Page 4 of 18

Assay Class: Eukaryote Total RNA Pico
Data Path: C:\...Eukaryote Total RNA Pico_DEDAE00458_2018-05-07_15-46-02.xad
Created: 5/7/2018 3:46:02 PM
Modified: 5/7/2018 4:13:29 PM

Electropherogram Summary

Ladder

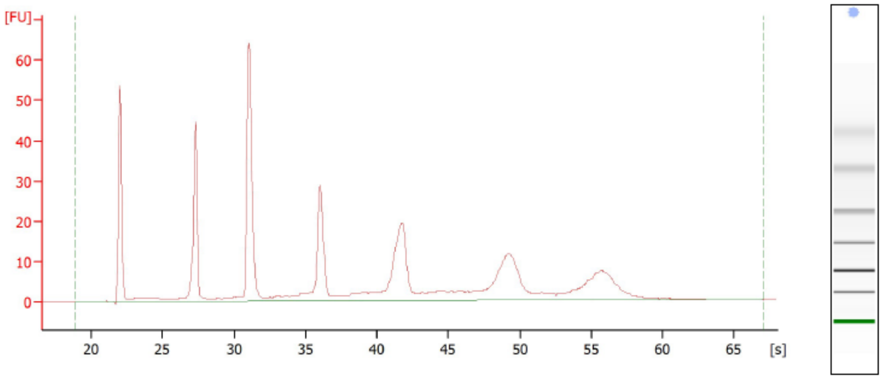

Overall Results for Ladder

RNA Area:	390.0	Result Flagging Color:	
RNA Concentration:	1,000 pg/µl	Result Flagging Label:	All Other Samples

2100 expert_Eukaryote Total RNA Pico_DEDAE00458_2018-05-07_15-46-02.xad Page 13 of 18

Assay Class: Eukaryote Total RNA Pico
Data Path: C:\...Eukaryote Total RNA Pico_DEDAE00458_2018-05-07_15-46-02.xad
Created: 5/7/2018 3:46:02 PM
Modified: 5/7/2018 4:13:29 PM

Electropherogram Summary Continued ...

Bull I

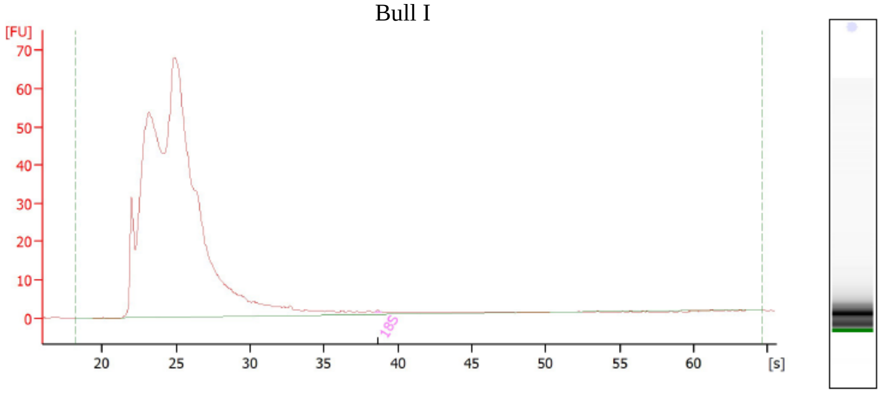

Overall Results for sample 9 : Bull I

RNA Area:	998.2	RNA Integrity Number (RIN):	2.5 (B.02.09)
RNA Concentration:	2,559 pg/µl	Result Flagging Color:	
rRNA Ratio [28s / 18s]:	0.0	Result Flagging Label:	RIN: 2.50

Fragment table for sample 9 : Bull I

**Name**	**Start Time [s]**	**End Time [s]**	**Area**	**% of total Area**
18S	38.43	38.85	0.3	0.0
